# Performance Analysis and Optimization of a Membrane Capacitive Deionization Process for Lithium Enrichment from Lithium-Ion Battery Wastewater

**DOI:** 10.3390/membranes16100324

**Published:** 2026-09-28

**Authors:** Minji Je, Seoyeon Lee, Juyoung Lee, Yongjun Choi, Sangho Lee

**Affiliations:** School of Civil and Environmental Engineering, Kookmin University, 77 Jeongneung-ro, Seongbuk-gu, Seoul 02707, Republic of Korea; jeminji123@kookmin.ac.kr (M.J.); yeon623@kookmin.ac.kr (S.L.); marks2@kookmin.ac.kr (J.L.); choiyj1041@kookmin.ac.kr (Y.C.)

**Keywords:** lithium recovery, membrane capacitive deionization, selectivity, secondary battery, alternative water resources

## Abstract

The rapid growth of lithium-ion battery (LIB) manufacturing has increased the generation of wastewater containing lithium and transition metals, creating a need for selective recovery processes with minimal chemical use. This study evaluated membrane capacitive deionization (MCDI) with a monovalent-selective cation-exchange membrane (MS-CEM) for lithium enrichment from simulated LIB manufacturing wastewater. One-factor-at-a-time experiments and response surface methodology were used to identify key operating parameters and optimize their interactions. Under the optimized conditions (1.43 V, pH 4.48, sorption time 500 s), the system achieved a lithium enrichment factor of 2.54 and a Li^+^/M^2+^ selectivity coefficient of 4.71. Over 100 cycles (approximately 28 h), these values decreased to 2.15 and 2.17, respectively. SEM–EDS detected Ni-, Co- and Mn-containing deposits on the spacer, electrodes and ion-exchange membranes, consistent with transition-metal fouling, although the deposit phases were not determined. A single cleaning with 0.01 M EDTA followed by 0.01 M HCl restored the selectivity coefficient to 4.38 and the enrichment factor to 2.25. These results indicate the potential of MS-CEM-assisted MCDI as a selective lithium pre-concentration step prior to hydrometallurgical recovery and identify selectivity loss, which coincided with transition-metal deposition, as the main limitation to sustained operation.

## 1. Introduction

The rapid expansion of lithium-ion battery (LIB) production for electric vehicles and portable electronics has been accompanied by increasing generation of wastewater from battery manufacturing and recycling processes [1,2]. Global LIB production capacity exceeded 500 GWh in 2022 and is projected to increase substantially over the coming decades as electric-vehicle deployment continues to accelerate [3,4]. This growth inevitably increases the discharge of wastewater containing lithium and other metal ions used in battery production. Unlike concentrated leachates generated during spent-battery recycling, wastewater from battery manufacturing is generally more dilute [5], making the recovery of valuable metals technically and economically challenging. Consequently, such wastewater represents both an environmental burden and a potential secondary source of critical materials.

LIBs contain lithium together with transition metals such as Ni, Co, and Mn in cathode materials including LiCoO_2_, LiMn_2_O_4_, LiNiMnCoO_2_, LiNiCoAlO_2_, and LiFePO_4_ [6]. During battery manufacturing and associated washing processes, a fraction of these metals can enter wastewater streams. Among them, lithium is particularly important because of its central role in rechargeable battery production and the rapidly increasing demand associated with energy-storage applications. Recovery of lithium from these dilute streams can therefore contribute to resource circularity while simultaneously reducing the metal load discharged from battery-manufacturing facilities [1,2,6]. However, selective lithium recovery is complicated by the simultaneous presence of divalent transition-metal ions such as Ni^2+^, Co^2+^, and Mn^2+^, which must be separated from Li^+^ before subsequent purification and recovery.

Conventional recovery of metals from LIB-related waste streams generally relies on hydrometallurgical processes. Transition metals are commonly removed by sequential pH adjustment and precipitation, followed by solvent extraction or other separation steps, whereas lithium is recovered from the remaining solution at a later stage [7,8,9]. Although these approaches can provide high recovery efficiencies for concentrated solutions, repeated addition of acids, bases, and other reagents can generate substantial quantities of secondary salts and chemical-laden wastewater [7,8,9,10,11]. These disadvantages become particularly important for dilute manufacturing wastewater, for which the chemical requirement per unit mass of recovered lithium can be relatively high. Therefore, a process capable of selectively separating and pre-concentrating lithium with limited reagent input before conventional hydrometallurgical treatment would be advantageous.

Membrane capacitive deionization (MCDI) offers a potential electrochemical approach for this purpose. MCDI removes ions by applying an electrical potential across porous electrodes while ion-exchange membranes control ionic transport between the solution and electrodes [12,13,14]. The process operates without high hydraulic pressure and can achieve adsorption and subsequent concentration of ions through cyclic charging and regeneration. However, conventional MCDI has limited intrinsic selectivity among cations [15,16,17,18,19]. Incorporation of ion-exchange membranes with ion-specific transport properties provides a means of overcoming this limitation. In particular, monovalent-selective cation-exchange membranes (MS-CEMs) can preferentially transport monovalent ions while restricting the transport of multivalent ions [15,20,21]. Thus, integrating an MS-CEM with MCDI may increase the Li^+^/M^2+^ ratio in the concentrated stream and enable MCDI to serve as a selective pre-concentration step upstream of hydrometallurgical lithium recovery.

The effectiveness of MCDI, however, depends strongly on operating conditions. Applied voltage controls the electrochemical driving force for ion transport, whereas feed pH can affect surface charge, ion speciation, and interactions with the ion-exchange membrane. Sorption time determines the extent to which differences in ion-transport kinetics can develop during a cycle, while flow rate and adsorption/desorption timing also influence mass transfer and concentration performance [22,23,24,25]. Because these parameters can interact nonlinearly, optimization based solely on one-factor-at-a-time (OFAT) experiments may fail to identify conditions that simultaneously maximize lithium enrichment and selectivity. Response surface methodology (RSM) provides a systematic approach for evaluating both individual and interaction effects using a statistically designed set of experiments and a polynomial response model [26,27,28,29]. RSM has therefore been increasingly applied to optimization of membrane and electrochemical separation processes in which multiple operating variables are interdependent [30].

Accordingly, this study systematically evaluates an eight-stack MCDI system equipped with a commercial MS-CEM for selective lithium enrichment from simulated dilute LIB-manufacturing wastewater containing Li^+^, Ni^2+^, Co^2+^, and Mn^2+^. The study was conducted in four stages. First, OFAT experiments were used to screen the effects of applied voltage, pH, sorption time, flow rate, and adsorption/desorption time ratio on the lithium enrichment factor and Li^+^/M^2+^ selectivity coefficient. Second, voltage, pH, and sorption time were further optimized using RSM to quantify their nonlinear and interaction effects and identify operating conditions that simultaneously enhance lithium enrichment and selectivity. Third, the optimized MCDI system was operated for 100 consecutive cycles to evaluate performance stability. Fourth, SEM–EDS analyses were used to identify metal deposition on electrodes, spacers, and ion-exchange membranes, and chemical cleaning was subsequently evaluated to determine the reversibility of the observed performance deterioration.

The novelty of this study lies in the integrated optimization and cyclic-performance assessment of MS-CEM-assisted MCDI for dilute LIB-manufacturing wastewater, rather than in the application of MCDI to lithium separation alone, which has been investigated previously [12,20]. Specifically, the study combines multivariable statistical optimization of lithium enrichment and Li^+^/divalent-metal selectivity with 100-cyclic operation, microscopic characterization of metal deposition, and evaluation of performance recovery after chemical cleaning. This integrated approach provides insight not only into the operating conditions required to enhance selective lithium transport but also into the factors that may limit sustained performance. The results therefore indicate the potential and practical limitations of MS-CEM-assisted MCDI as a selective pre-concentration step for downstream lithium recovery from dilute LIB-manufacturing wastewater.

## 2. Materials and Methods

### 2.1. Feed Water

Synthetic wastewater was prepared to represent dilute wastewater generated during the manufacturing of lithium-ion battery (LIB) cathode materials. Commercial NCM622 cathode materials have a nominal stoichiometric metal ratio of Li^+^:Ni^2+^:Co^2+^:Mn^2+^ = 10:6:2:2 [31,32,33,34]. During cathode manufacturing, however, lithium is generally added in excess to facilitate complete reaction with transition metals [35]. A fraction of this excess lithium remains unreacted and is subsequently discharged during washing processes [36,37]. Accordingly, the synthetic wastewater used in this study was prepared with a higher relative lithium content, corresponding to a Li^+^:Ni^2+^:Co^2+^:Mn^2+^ molar ratio of 10:3:1:1.

LiCl, NiSO_4_·6H_2_O, CoSO_4_·7H_2_O, and MnSO_4_·H_2_O (Sigma-Aldrich, St. Louis, MO, USA) were used as the sources of Li^+^, Ni^2+^, Co^2+^, and Mn^2+^, respectively. The total salt concentration was approximately 900 mg/L to represent the relatively dilute characteristics of wastewater generated from LIB cathode manufacturing processes [5]. The feed composition is summarized in Table 1. The corresponding concentrations of Li^+^, Ni^2+^, Co^2+^, and Mn^2+^ were approximately 37.6, 95.3, 31.9, and 29.7 mg/L, respectively. The pH of the feed solution was adjusted to the target value before each experiment using H_2_SO_4_.

Because H_2_SO_4_ introduces sulfate in addition to that supplied by NiSO_4_, CoSO_4_ and MnSO_4_, the additional anion load was evaluated. The three metal sulfate salts contribute 2.71 mmol/L (260 mg/L) of sulfate. The acid volumes added at each pH condition were not recorded, so the total sulfate concentration at each condition could not be determined directly. A lower bound can nevertheless be estimated from the free H^+^ concentration at the target pH, because buffering by metal hydrolysis species and the formation of HSO_4_^−^ can only increase the acid demand. On this basis, acid addition contributed at least 0.5, 1.6, 4.8, 48 and 230 mg/L of sulfate at pH 5, 4.48, 4, 3 and 2.32, respectively, corresponding to at least 0.2%, 0.6%, 1.8%, 18% and 88% of the sulfate supplied by the metal salts (Supplementary Section S5). The estimates based on the target pH do not establish that sulfate addition was negligible at the optimized condition. Consequently, the observed pH dependence may also reflect accompanying changes in sulfate concentration, ionic strength, and metal speciation. These contributions could not be separated from the effect of pH in the present study (Section 3.5).

H_2_SO_4_ was used in preference to HCl in order to preserve a sulfate-dominated anion matrix representative of cathode-manufacturing wastewater, in which sulfate is the principal anion; HCl would have avoided the additional sulfate but would have introduced additional chloride, and the two acids were not compared experimentally. Sulfate also forms neutral ion pairs with Ni^2+^, Co^2+^ and Mn^2+^, whereas the association of Li^+^ with sulfate is much weaker. Because neutral ion pairs are not driven by electromigration, the measured selectivity coefficients may reflect the combined effect of membrane monovalent selectivity and solution-phase speciation rather than membrane properties alone (Section 3.5). The total salt concentration of approximately 900 mg/L refers to the mass of dissolved salts added and does not include the acid.

### 2.2. MCDI Module

Membrane capacitive deionization (MCDI) removes ions by applying an electrical potential across porous electrodes separated from the feed channel by ion-exchange membranes [17,38,39,40,41,42]. As shown in Figure 1, cations and anions migrate toward oppositely charged electrodes and are stored within the electrical double layers of the electrodes during the deionization cycle. Reversal of the applied potential releases the adsorbed ions, producing an ion-enriched concentrate stream during regeneration. An eight-stack MCDI module was used in this study to provide sufficient electrode area for ion adsorption and subsequent concentration [43,44]. Each repeating unit consisted of activated-carbon electrodes, ion-exchange membranes, and spacers arranged to form alternating flow channels, as illustrated in Figure 2.

A commercial monovalent-selective cation-exchange membrane (MS-CEM; ASTOM Corporation, Tokyo, Japan) was incorporated into the MCDI module to preferentially transport Li^+^ while restricting the transport of divalent transition-metal ions. A standard non-selective CEM was also used for comparison during preliminary membrane testing. The major components and dimensions of the MCDI module are summarized in Table 2. The monovalent-selective cation-exchange membrane carries a positively charged surface layer applied to a styrene–divinylbenzene base membrane, whereas the standard CEM and AEM are styrene–divinylbenzene membranes with a reinforcing fabric [45]. The experiments were performed under identical operating conditions using the standard CEM and MS-CEM configurations to compare their monovalent-ion removal performance. Based on this preliminary evaluation, the MS-CEM configuration was used in all subsequent lithium-selectivity experiments.

The surface area (BET), pore size dispersion, specific capacitance, and binder content of the activated carbon electrodes were not disclosed by the manufacturer, which regards this information as proprietary, and were not measured independently in this study. Consequently, their individual contributions to ion adsorption and cycling performance could not be determined. The reported performance therefore applies to the tested electrode configuration, and direct comparison or reproduction using alternative electrodes requires consideration of this characterization limitation.

### 2.3. Experimental Setup of MCDI Process

The laboratory-scale MCDI system consisted of a feed reservoir, valve, pump, eight-stack MCDI module, DC power supply, and separate vessels for the treated and concentrate streams, as illustrated in Figure 1 and Figure 2. Feed solution was supplied to the MCDI module at controlled flow rates of 10–30 mL/min, depending on the experimental condition, using a pump. The electrical potential was applied using a power supply. During the adsorption step, a positive potential was applied across the electrodes, causing ions in the feed solution to migrate through the ion-exchange membranes and accumulate at the electrodes. The effluent discharged during this period was collected as the treated stream. During the subsequent desorption step, the electrode polarity was reversed, releasing the previously adsorbed ions. The desorption effluent was collected separately as the concentrate stream and analyzed for Li^+^, Ni^2+^, Co^2+^, and Mn^2+^.

### 2.4. One-Factor-at-a-Time Experiments

One-factor-at-a-time (OFAT) experiments were initially conducted to screen the effects of operating conditions on lithium enrichment and selectivity. Five variables were investigated: applied voltage (1.2–1.6 V), feed pH (3–5), adsorption/desorption time (300–500 s), flow rate (10–30 mL/min), and adsorption/desorption time ratio (1:0.8–1:1.2). For each OFAT experiment, only one variable was varied while all other variables were maintained at their reference conditions. The reference conditions were voltage = 1.4 V, pH = 4, adsorption/desorption time = 400 s, flow rate = 10 mL/min, and time ratio = 1:1. Each condition was tested at least three times to ensure reliable results. MCDI performance was evaluated using the lithium enrichment factor (EF) and Li^+^/M^2+^ selectivity coefficient (SC), where M^2+^ represents the competing divalent ions Ni^2+^, Co^2+^, and Mn^2+^. The OFAT results were used to select the variables and operating ranges for subsequent multivariable optimization.

### 2.5. Response Surface Methodology

Response surface methodology (RSM) was subsequently applied to quantify nonlinear and interaction effects among the principal operating variables and to identify conditions that simultaneously enhanced lithium enrichment and selectivity. Based on the OFAT screening results and the electrochemical relevance of the variables, applied voltage (A), feed pH (B), and sorption time (C) were selected as independent factors. Flow rate was fixed at 10 mL/min because this condition provided the highest overall EF and SC during OFAT testing. The adsorption/desorption time ratio was fixed at 1:1 during the RSM experiments. To ensure reliable and reproducible measurements, the membranes and electrodes used during the OFAT experiments were replaced with new components before initiating the RSM experiments. The same set of membranes and electrodes was then used throughout the RSM experiments, with appropriate washing and cleaning between experimental runs.

A central composite design (CCD) was employed using five levels for each factor. The axial distance was α = 1.68179. The design comprised 20 experimental runs: eight factorial points, six axial points, and six replicates at the center point. The center-point condition was 1.4 V, pH 4, and 400 s, and the six center-point replicates were used to estimate pure experimental error. The experimental responses were fitted to a second-order polynomial model. Model significance was evaluated by analysis of variance (ANOVA) using the F-statistic and associated *p*-values. Terms with *p* < 0.05 were considered statistically significant. Model adequacy was evaluated using the coefficient of determination (R^2^), residual diagnostics, and lack-of-fit test. The factors and responses are summarized in Table 3.

A simultaneous numerical optimization was subsequently performed with EF and SC specified as responses to be maximized, using the desirability-function approach implemented in Design-Expert (version 13, Stat-Ease Inc., Minneapolis, MN, USA). Both responses were assigned the goal “maximize” over the ranges observed in the design (EF 1.44–2.86; SC 1.39–3.56), and all three factors were constrained to their factorial ranges (1.2–1.6 V, pH 3–5, 300–500 s) in order to avoid extrapolation towards the axial conditions. Individual desirability functions were defined as linear (weight = 1) between the lower and upper response limits, and the two responses were assigned equal importance. The overall desirability was calculated as the geometric mean of the individual desirabilities, and the solution with the highest overall desirability was adopted as the optimized condition used for cyclic operation testing. The desirability calculation was reproduced retrospectively from the fitted models (Supplementary Section S4, Tables S5 and S6); this reanalysis is distinct from the original Design-Expert computation but located the equal-weight optimum close to the tested condition.

Model adequacy was additionally assessed using the predicted coefficient of determination obtained from the PRESS statistic, leave-one-out cross-validation, the adequate-precision signal-to-noise ratio, and the coefficient of variation (Supplementary Table S1); residual diagnostic plots (normal probability plot of internally studentized residuals, residuals versus predicted values, residuals versus run label, and predicted versus actual values) and influence statistics (leverage and Cook’s distance) are provided in the Supplementary Materials (Section S3, Table S4 and Figures S1 and S2).

### 2.6. Cyclic Operation and Chemical Cleaning

The operating condition identified through simultaneous RSM optimization was first experimentally validated. The selected condition was 1.43 V, pH 4.48, and a sorption time of 500 s, with the flow rate maintained at 10 mL/min and the adsorption/desorption time ratio maintained at 1:1. A 10-cycle validation experiment was first conducted under these conditions to verify the predicted improvement in EF and SC. Subsequently, the MCDI system was operated continuously for 100 adsorption/desorption cycles under the same conditions to evaluate performance stability over repeated cycling. During the cyclic operation, the feed was supplied to the module only during the adsorption step. The desorption effluent was circulated through a concentrate loop (total volume 300 mL, initially filled with deionized water), which was sampled and renewed after every 10 cycles. Then, samples were collected every 10 cycles, and EF and SC were monitored as indicators of performance deterioration. The complete test corresponds to approximately 28 h of continuous operation.

After the 100-cycle test, the module was disassembled and the spacer, electrodes and ion-exchange membranes were examined by SEM–EDS to characterize accumulated deposits (Section 3.3.2). A two-step chemical cleaning procedure was then applied. First, 0.01 M disodium ethylenediaminetetraacetate (Na_2_EDTA; CAS 139-33-3, Sigma-Aldrich) was used as a chelating agent to remove accumulated transition-metal species. This was followed by 0.01 M HCl to remove residual inorganic deposits. The cleaning was performed by circulating the cleaning solution in place, followed by deionized water rinsing. The cleaning and rinsing were conducted at room temperature (25 °C). After the cleaning, the same components were subsequently reassembled for the performance evaluation.

### 2.7. Performance Indicators

MCDI performance was evaluated using ion removal efficiency, the lithium enrichment factor, the Li^+^/M^2+^ selectivity coefficient, lithium recovery, and the lithium molar fraction of the concentrate. The ion removal efficiency (*R_i_*) during adsorption was calculated as:

(1) $$R_{i}\left(\%\right)=\frac{C_{i,feed}-\;C_{i,treated}}{C_{i,feed}}\times 100\;$$

where *C_i_*_,*feed*_ and *C_i_*_,*treated*_ are the concentrations of ion (*i*) in the feed and treated streams, respectively.

The lithium enrichment factor was calculated as:

(2) $${EF}_{Li}=\frac{C_{Li,f}}{C_{Li,0}}$$

where *C_li_*,_0_ is the Li^+^ concentration in the feed solution and *C_li_*,*_f_* is the Li^+^ concentration in the desorption concentrate. An EF greater than 1 indicates lithium enrichment relative to the initial feed.

Lithium selectivity relative to the competing divalent ions was calculated as:

(3) $${SC}_{M^{2+}}=\frac{\frac{C_{Li,f}}{C_{Li,0}}}{\frac{C_{M^{2+},f}}{C_{M^{2+},0}}}=\frac{{EF}_{Li}}{{EF}_{M^{2+}}}$$

where *C_M_*^2+^_,0_ and *C_M_*^2+^_,*f*_ are the initial and final concentrations, respectively, of Ni^2+^, Co^2+^, and Mn^2+^. Thus, the average *SC* is given by:

(4) $$\;{SC}_{avg}=\frac{{EF}_{Li}}{\frac{1}{3}\sum_{j}{EF}_{M^{2+}}}=\frac{3{EF}_{Li}}{{EF}_{{Ni}^{2+}}+{EF}_{{Co}^{2+}}+{EF}_{{Mn}^{2+}}}=\frac{3}{\frac{1}{{SC}_{{Ni}^{2+}}}+\frac{1}{{SC}_{{Co}^{2+}}}+\frac{1}{{SC}_{{Mn}^{2+}}}}$$

Equation (4) defines the average selectivity coefficient as the lithium enrichment factor divided by the arithmetic mean of the enrichment factors of the three divalent metals. This is algebraically equivalent to the harmonic mean of the three pairwise selectivity coefficients given by Equation (3). The definition was adopted so that the average is weighted towards the least effectively rejected divalent species, rather than being dominated by the most effectively rejected one; consequently, the reported averages are lower than the arithmetic means of the pairwise coefficients.

Lithium recovery was defined as the mass of Li^+^ collected in the concentrate stream divided by the mass of Li^+^ supplied in the feed over the complete adsorption–desorption cycle, and the lithium molar fraction of the concentrate was defined as the molar amount of Li^+^ divided by the total molar amount of Li^+^, Ni^2+^, Co^2+^ and Mn^2+^ in the concentrate. The latter quantity is reported because it expresses the degree of purification achieved relative to the feed composition, which the enrichment factor alone does not convey.

The charge efficiency (*CE*) is defined as:

(5) $$CE=\frac{F\sum_{i}z_{i}∆n_{i}}{Q_{ads}}$$

where *F* is the Faraday’s constant; *Q_ads_* is the charge passed; *z_i_* is the ion valency; and Δ*n_i_* is the quantity of the removed ions. The energy consumption per mole of Li^+^ collected in the concentrate (*SEC*) can be defined as:

(6) $$SEC=\frac{\frac{1}{3.6\times 10^{6}}\int_{0}^{t_{cycle}}P_{in}(t)dt}{\frac{c_{Li,c}V_{c}}{1000{MW}_{Li}}}$$

where *t_cycle_* is the time for one cycle, *P_in_*(*t*) is the electrical power supplied to the module (W); *c_Li_*_,*c*_ is the volume-weighted Li^+^ concentration in the concentrate (mg/L); *V_c_* is the collected concentrate volume (L); and *MW_Li_* = 6.94 g/mol. *Q_ads_ and P_in_* refer to the whole stack, and desorption energy was subtracted to obtain a net value.

### 2.8. Analytical Methods

The electrical conductivity of the feed and effluent streams was monitored using a conductivity meter (WTW Multi 3620 IDS, Tetracon 925 cell, Tetracon, Islamabad, Pakistan). The concentrations of Li^+^, Ni^2+^, Co^2+^, and Mn^2+^ in the feed, in the treated stream collected during the adsorption step, and in the concentrate were determined by inductively coupled plasma atomic emission spectrometry (ICP-AES; IRIS Intrepid, Thermo Elemental, Franklin, MA, USA). Each sample was analyzed in triplicate, and the mean values were used to calculate the ion removal efficiency (Equation (1)), the enrichment factor (Equation (2)), the selectivity coefficients (Equations (3) and (4)), and the lithium recovery.

Surface morphology was examined by field-emission scanning electron microscopy (FE-SEM; SU8700, Hitachi High-Tech, Tokyo, Japan), and elemental composition was determined using energy-dispersive X-ray spectroscopy (JSM-7160F, JEOL Ltd., Tokyo, Japan). The SEM-EDS measurements were used to characterize the location and elemental composition of deposits accumulated on individual MCDI components during repeated operation. EDS is a semi-quantitative technique, and the weight percentages reported in Section 3.3.2 are subject to appreciable uncertainty arising from surface roughness, the small thickness of the deposits relative to the electron interaction volume, and the limited accuracy of standardless quantification. They are therefore used for relative comparison among components rather than as absolute surface compositions, and no crystallographic or chemical-state analysis of the deposits was performed. EDS spectra were acquired at a single location on each component, so the results describe the analyzed locations and do not provide statistically representative comparisons between entire components.

## 3. Results and Discussion

### 3.1. OFAT Experiments

One-factor-at-a-time (OFAT) experiments were conducted to examine the individual effects of applied voltage, sorption time, flow rate, feed pH, and adsorption/desorption time ratio on lithium enrichment and selectivity. The enrichment factor (EF) and Li^+^/M^2+^ selectivity coefficient (SC), where M^2+^ represents Ni^2+^, Co^2+^, and Mn^2+^, are presented in Figure 3 and Figure 4, respectively. The ion-specific selectivity coefficients are summarized in Table 4. These OFAT experiments were intended to provide the basis for selecting key variables as the factors for subsequent response surface methodology analysis.

#### 3.1.1. Effect of Operating Conditions on Enrichment Factor

Figure 3 shows the effects of the five operating variables on the lithium enrichment factor. Increasing the voltage from 1.2 to 1.4 V increased EF from 1.55 to 1.65, whereas a further increase to 1.6 V resulted in a slightly lower EF of 1.63. Thus, voltage enhanced lithium enrichment only within a limited range, and increasing the electrical driving force alone was insufficient to continuously improve concentration performance. Several studies report similar behaviors: ion removal drops quickly at first, then slows or levels off as adsorption sites approach saturation [46,47]. The result suggests that an intermediate voltage provided a more favorable balance between lithium electrosorption and the simultaneous transport of competing ions.

Sorption time had a more pronounced effect. Increasing the adsorption/desorption time from 300 to 400 and 500 s increased EF from 1.43 to 1.65 and 1.76, respectively. The longer cycle provided more time for lithium transport and accumulation within the electrodes and subsequently increased the lithium concentration released during desorption. Among the individual variables examined over the selected ranges, sorption time produced the clearest improvement in EF.

In contrast, flow rate had little effect on lithium enrichment. EF decreased only slightly from 1.63 at 10 mL/min to 1.62 and 1.60 at 20 and 30 mL/min, respectively. This limited variation indicates that, within the investigated range, lithium enrichment was not strongly controlled by bulk flow rate. Nevertheless, a slightly higher EF was obtained at 10 mL/min, together with its favorable selectivity.

Feed pH produced a gradual but moderate increase in EF. The enrichment factor increased from 1.45 at pH 3 to 1.54 at pH 4 and 1.62 at pH 5. This result shows that the chemical environment of the feed affected electrosorption and membrane transport, although its influence was weaker than that of sorption time. Because metal speciation, membrane charge, and electrode surface chemistry were not measured directly, the pH effect should be interpreted as an empirical process response rather than as evidence for a specific separation mechanism.

The adsorption/desorption time ratio had a different effect from the absolute sorption time. The highest EF of 1.82 was obtained at a ratio of 1:0.8, whereas EF decreased to 1.63 at 1:1 and 1.58 at 1:1.2. A shorter desorption period can produce a more concentrated effluent by releasing the accumulated ions into a smaller volume. However, the resulting increase in EF does not necessarily indicate greater lithium recovery or improved selectivity. This distinction is important because the time ratio that maximized EF did not maximize SC.

In summary, the OFAT results show that lithium enrichment could be moderately improved by extending sorption time or reducing the relative desorption duration. However, the maximum EF achieved by single-factor adjustment was limited to 1.82, indicating that optimization based solely on individual variables was insufficient to achieve a substantial improvement in performance.

#### 3.1.2. Effect of Operating Conditions on Li Selectivity

Figure 4 presents the effect of the operating variables on the Li^+^/M^2+^ selectivity coefficient. Voltage produced a modest increase in SC, from 1.59 at 1.2 V to 1.65 at 1.4 V and 1.85 at 1.6 V. The gradual increase indicates that a stronger electrical driving force moderately favored lithium transport relative to the divalent metals. However, the small magnitude of the change confirms that voltage alone was not the dominant factor controlling selectivity.

Sorption time had the strongest individual effect on SC. Increasing the cycle duration from 300 to 400 s increased SC from 1.31 to 1.65, and a further increase to 500 s raised SC to 2.40. The stronger time dependence of SC compared with EF suggests that lithium and the competing divalent ions exhibited different transport kinetics through the MS-CEM-assisted MCDI system. Longer sorption periods allowed these kinetic differences to become more pronounced, thereby improving the separation of Li^+^ from Ni^2+^, Co^2+^, and Mn^2+^.

Increasing the flow rate reduced selectivity. SC decreased from 1.65 at 10 mL/min to 1.40 at 20 mL/min and 1.30 at 30 mL/min. Although flow rate had little effect on EF, its influence on SC indicates that the relative transport of lithium and divalent ions was sensitive to hydraulic contact time. The shorter residence time at higher flow rates may have reduced the extent to which the MS-CEM differentiated between ions with different transport characteristics. Accordingly, 10 mL/min was selected as the fixed flow rate for the RSM experiments.

The effect of feed pH was non-monotonic. SC increased from 1.53 at pH 3 to a maximum of 1.65 at pH 4 and then decreased slightly to 1.55 at pH 5. This result indicates that a moderately acidic condition was favorable for selective lithium transport. However, because the present experiments did not measure membrane charge, local pH changes, or dissolved-metal speciation, the specific origin of the pH dependence cannot be determined from the current data.

Unlike EF, SC increased as the desorption period became longer relative to the adsorption period. SC increased from 1.35 at a time ratio of 1:0.8 to 1.65 at 1:1 and 1.81 at 1:1.2. The opposite responses of EF and SC demonstrate an operational trade-off. A shorter desorption period increased the concentration of lithium in the collected stream, whereas a longer desorption period improved the relative separation of lithium from the divalent metals. The adsorption/desorption time ratio was therefore not selected as an independent RSM factor because its effect was closely related to effluent volume and because it did not simultaneously maximize EF and SC.

The ion-specific results in Table 4 provide further information on competitive transport. Increasing the voltage from 1.2 to 1.6 V increased the Li^+^/Ni^2+^ selectivity coefficient from 1.12 to 1.34 and the Li^+^/Co^2+^ coefficient from 1.57 to 1.94, whereas Li^+^/Mn^2+^ selectivity varied non-monotonically between 2.17 and 2.62. Sorption time produced a stronger and more consistent response. At 500 s, the Li^+^/Ni^2+^, Li^+^/Co^2+^, and Li^+^/Mn^2+^ selectivity coefficients reached 1.82, 2.54, and 3.24, respectively. In contrast, increasing the flow rate from 10 to 30 mL/min reduced these coefficients from 1.52, 1.68, and 1.77 to 1.27, 1.30, and 1.33, respectively. Their convergence at the highest flow rate indicates that shorter hydraulic contact time reduced the differentiation among the ion transport rates. Li^+^/Ni^2+^ selectivity was generally the lowest among the three ion pairs, indicating that Ni^2+^ was the most difficult divalent metal to separate from lithium under the tested conditions.

Overall, the OFAT experiments identified sorption time as the most influential individual factor for Li^+^/M^2+^ selectivity. Nevertheless, the maximum SC of 2.40 remained moderate, demonstrating the need for multivariable optimization to account for nonlinear effects and interactions among voltage, pH, and sorption time.

Among the three ion pairs, the Li^+^/Ni^2+^ selectivity coefficient was consistently the lowest, reaching 1.82 at the most favorable OFAT condition. Two factors may contribute to this behavior. First, Ni^2+^ has the smallest hydrated radius of the three divalent metals considered (Ni^2+^ ≈ 0.404 nm, Co^2+^ ≈ 0.423 nm, Mn^2+^ ≈ 0.438 nm) [48], and if exclusion by the positively charged, densely cross-linked surface layer of a monovalent-selective cation-exchange membrane is governed largely by hydrated size, discrimination would be least effective for Ni^2+^ [45,49]. Hydration energy does not explain the ordering: its magnitude increases in the order Mn^2+^ < Co^2+^ < Ni^2+^ [50], which would favor the exclusion of Ni^2+^ if dehydration controlled entry into the selective layer. Second, Ni^2+^ was present at three times the molar concentration of Co^2+^ and Mn^2+^ in the simulated feed. Because the selectivity coefficient is normalized to the feed concentration, this difference does not by itself lower SC; however, competitive uptake in the selective layer need not scale linearly with feed concentration, so a concentration effect cannot be excluded.

The ordering of the pairwise coefficients in Table 4 is consistent with the trend in hydrated radius, but the present data cannot separate the two factors. For context, the monovalent/divalent selectivity reported for commercial monovalent-selective cation-exchange membranes varies widely, from approximately 1.2 to 50 depending on solution composition, current density and cell configuration [49], and an MCDI system employing the same class of membrane for Li^+^/Mg^2+^ separation reported a lithium selectivity coefficient of 2.95 [20]. The values obtained here for a three-component divalent mixture therefore fall within the lower part of the range reported for commercial monovalent-selective membranes, and the low Li^+^/Ni^2+^ coefficient remains a significant practical limitation (Section 3.5).

### 3.2. Response Surface Methodology

A central composite design consisting of 20 experimental runs was used to evaluate the combined effects of applied voltage, feed pH, and sorption time. The experimentally measured EF values ranged from 1.44 to 2.86, whereas SC ranged from 1.39 to 3.56. The six center-point experiments produced EF values of 2.35–2.48 and SC values of 2.75–2.99. The corresponding standard deviations were 0.055 and 0.106, respectively, indicating greater center-point variability for SC than for EF. Reduced quadratic response models were developed for both responses and evaluated using analysis of variance.

The OFAT and RSM experiments were conducted as separate experimental blocks using component sets with different operating histories. The OFAT screening was performed using previously operated membranes and electrodes, whereas a new set was installed before the CCD experiments and used throughout the RSM runs. Accordingly, absolute EF and SC values obtained in the two stages are not directly comparable. The difference was large: at the common condition of 1.4 V, pH 4 and 400 s, the OFAT experiments gave EF = 1.54–1.65 and SC = 1.65, whereas the six CCD center points gave EF = 2.35–2.48 and SC = 2.75–2.99. Component condition therefore strongly affected absolute performance, and absolute optima identified with one component set may not transfer to another (Section 3.3.1). The OFAT results were therefore used primarily to identify qualitative factor trends and operating ranges, whereas the performance of the selected RSM condition was assessed relative to the RSM model and independent validation experiments.

#### 3.2.1. RSM Results: Enrichment Factor

The measured EF values and corresponding ANOVA results are presented in Table 5 and Table 6. The highest experimental EF within the CCD matrix was 2.86 at 1.4 V, pH 4, and a sorption time of 568 s. A similarly high EF of 2.73 was obtained at the same voltage and pH with a sorption time of 232 s. In contrast, the lowest EF of 1.44 was observed at 1.06 V, pH 4, and 400 s. These results indicate that EF exhibited substantial curvature across the experimental domain rather than a simple monotonic response to each factor.

After removal of non-significant terms, the coded quadratic equation for EF was expressed as:

Y_1_ = 2.4155 + 0.1255A + 0.0313B + 0.0211C + 0.0613BC − 0.2281A^2^ − 0.1627B^2^ + 0.1308C^2^ (7)

where Y_1_ is the predicted enrichment factor and A, B, and C are the coded values of voltage, pH, and sorption time, respectively. The reduced model was statistically significant (F = 30.82, *p* < 0.0001) and explained 94.7% of the observed variation (R^2^ = 0.9473; adjusted R^2^ = 0.9166; predicted R^2^ = 0.778; coefficient of variation 3.97%; adequate precision 22.4). The linear voltage term was significant (*p* = 0.0002), whereas the linear effects of pH (*p* = 0.2182) and sorption time (*p* = 0.3968) were not significant. The quadratic terms for voltage, pH, and sorption time were all significant. The pH–sorption time interaction did not reach the conventional significance criterion but showed a marginal effect within the investigated range (*p* = 0.0750).

These statistical results show that EF was governed primarily by nonlinear curvature rather than by strong synergistic interactions among the three factors. The negative coefficient of A^2^ indicates that EF was maximized at an intermediate voltage and decreased toward both the low- and high-voltage limits. The negative B^2^ coefficient similarly indicates a favorable intermediate pH region. In contrast, the positive C^2^ coefficient reflects a U-shaped response over the CCD range, with the two axial sorption-time conditions producing higher EF values than the center-point duration. At first sight this appears to contradict the OFAT results, which showed a monotonic increase in EF between 300 and 500 s, and the apparent discrepancy therefore requires explanation. Evaluated at the OFAT reference voltage and pH (1.4 V, pH 4), the reduced model predicts EF values of 2.53, 2.42 and 2.57 at 300, 400 and 500 s, respectively (Supplementary Table S6), compared with 1.43, 1.65 and 1.76 in the OFAT experiments. The model therefore agrees with the OFAT data only in the net change between 300 and 500 s; it retains an intermediate minimum that the OFAT data do not show, so the two data sets are not fully reconciled. The intermediate minimum amounts to only 0.11–0.15 EF units, which corresponds to 1.2–1.7 residual standard deviations (residual standard deviation 0.089; pure-error standard deviation at the centre point 0.055). The curvature within the 300–500 s interval is therefore weakly resolved, and the U-shaped form of the fitted surface is driven predominantly by the two axial conditions at 232 and 568 s.

One possible contribution to the elevated EF at the shortest sorption time is the dependence of EF on the volume into which the desorbed ions are released. Because the adsorption/desorption time ratio was fixed at 1:1, reducing the sorption time also reduces the volume of concentrate collected during the desorption step. As EF is a concentration ratio, a smaller accumulated ion inventory released into a proportionally smaller volume can still yield a high enrichment factor without indicating improved separation, provided that ion uptake per unit time is highest early in the adsorption step. A related effect was observed in the OFAT experiments, in which the shortest relative desorption period (time ratio 1:0.8) produced the highest EF (1.82) together with the lowest selectivity coefficient (1.35). However, in the CCD the adsorption and desorption durations changed together, whereas in the OFAT ratio series only the desorption duration changed, so this comparison is indicative rather than direct. Consistent with this interpretation, the SC model predicts a value of only 2.43 at 232 s compared with 3.36 at 500 s, so the short-time enrichment maximum is not accompanied by selective lithium transport. At longer sorption times the increase in the accumulated ion inventory may become dominant and EF rises again. These two contributions may account for the curvature of the fitted surface, although the difference in component condition between the OFAT and CCD stages was not isolated experimentally. The simultaneous optimization selected the long-sorption-time branch, where both modeled responses are favorable.

The response surfaces in Figure 5 visualize these nonlinear effects. Pronounced curvature was observed in the voltage-related surfaces, confirming the importance of selecting an appropriate intermediate voltage. The pH-related surfaces also show a favorable enrichment region near the center of the investigated pH range. Within the factorial operating range, the reduced model identified a candidate high-EF region near approximately 1.46 V, pH 4.29, and 500 s.

The lack-of-fit test for the reduced EF model was not significant at the 5% level (F = 3.75, *p* = 0.0822). This result supports the adequacy of the model for describing EF within the investigated factor range. However, because the *p*-value was relatively close to 0.05 and the response showed pronounced curvature at the axial conditions, the model should be used primarily to identify a favorable local operating region rather than for extrapolation beyond the experimental domain.

#### 3.2.2. RSM Results: Li Selectivity

The second RSM response was the Li^+^/M^2+^ selectivity coefficient rather than a mass-based lithium recovery ratio. The highest experimental SC in the CCD matrix was 3.56 at 1.4 V, pH 4, and 568 s, whereas the lowest value of 1.39 was obtained at 1.4 V, pH 2.32, and 400 s. The low SC at the acidic axial point and the higher values at moderate pH and extended sorption time demonstrate that both pH and cycle duration influenced ion-selective transport.

The reduced coded equation for SC was as follows, and the corresponding ANOVA results are presented in Table 7:

Y_2_ = 2.8717 − 0.0554A + 0.1917B + 0.2300C + 0.4013BC − 0.3654A^2^ − 0.5033B^2^ + 0.1084C^2^ (8)

where Y_2_ is the predicted Li^+^/M^2+^ selectivity coefficient. The reduced SC model was statistically significant (F = 38.81, *p* < 0.0001) and explained 95.77% of the measured variation (R^2^ = 0.9577; adjusted R^2^ = 0.9330; predicted R^2^ = 0.820; coefficient of variation 7.37%; adequate precision 22.2). The linear effects of pH and sorption time were significant, with *p* = 0.0015 and *p* = 0.0004, respectively, whereas the linear voltage effect was not significant (*p* = 0.2604).

Among the interaction terms, the pH–sorption time interaction was significant (*p* < 0.0001). Thus, the influence of sorption time on lithium selectivity depended strongly on feed pH, whereas voltage influenced SC primarily through its significant quadratic term rather than through a linear effect. The quadratic terms for voltage and pH were highly significant (*p* < 0.0001), and the quadratic sorption-time term was also significant (*p* = 0.0352). The negative A^2^ coefficient indicates that selectivity was favored at an intermediate voltage rather than at either voltage extreme.

The pH response also exhibited a pronounced maximum because both the positive linear pH term and negative quadratic pH term were significant. The positive *BC* coefficient shows that the benefit of extending sorption time became stronger under favorable pH conditions. This statistical interaction is consistent with the response surfaces in Figure 6, which show the highest selectivity region at moderately acidic pH and relatively long sorption time.

The strong sorption-time dependence is also consistent with the OFAT results. Extending the cycle provides more time for differences between lithium and divalent-ion transport to develop. However, the observed pH–time interaction demonstrates that longer operation alone is insufficient; the chemical environment must also be appropriate for the MS-CEM to maintain preferential lithium transport.

Within the factorial operating range, the reduced model identified a high-SC region near approximately 1.39 V, pH 4.59, and a sorption time of 500 s. The lack-of-fit test was not significant at the 5% level (F = 3.87, *p* = 0.0776), supporting use of the model to describe local trends and factor importance. As the lack-of-fit probability remained close to 0.05, quantitative predictions should nevertheless be verified experimentally, particularly near the boundaries of the design space.

#### 3.2.3. Consideration of Faradaic Side Reactions

Because the investigated voltage range extended above the standard thermodynamic potential for water splitting (1.23 V), the possibility of faradaic side reactions warrants explicit consideration. The applied cell voltage is distributed across the two electrode/electrolyte interfaces, the two ion-exchange membranes, the spacer channel and the dilute bulk solution, so the interfacial overpotential at each electrode is appreciably lower than the applied cell voltage. In combination with the large kinetic overpotentials for hydrogen and oxygen evolution on activated carbon, this widens the practical operating window relative to the thermodynamic limit [51]. Cell voltages in the range 1.2–1.6 V are accordingly used routinely in CDI and MCDI studies, and operation above 1.23 V has been applied deliberately where a higher ion-transport driving force is required. In a flow-electrode CDI system, charge efficiency decreased gradually from 92% to 69.5% as the cell voltage was raised from 0.6 V to 4.8 V [52]; because the flow-electrode configuration differs substantially from the fixed-electrode module used here, this result shows only that the loss need not be abrupt and cannot be transferred quantitatively to the present system. Ion-exchange membranes further suppress parasitic reactions relative to membrane-free CDI by restricting co-ion access to the electrode region [51].

Both response models contain a significant negative quadratic voltage term (A^2^ = −0.2281 for EF and A^2^ = −0.3654 for SC, both *p* < 0.0001), and both EF and SC decreased at the highest axial voltage of 1.74 V. This behavior is compatible with an increasing faradaic contribution at higher voltages, but response-surface curvature alone cannot identify its cause or magnitude. The low charge efficiency estimated at the optimized condition (7.6–8.7%; Section 3.4), and faradaic reactions may account for part of this deficit. Charge efficiency was not determined across the voltage range, and coulombic efficiency, effluent pH and gas evolution were not monitored. The condition of 1.43 V is therefore interpreted as an empirical operating point that maximized the modeled concentration responses within the investigated range, not as a demonstrated electrochemically stable or energy-optimal voltage; this is identified as a limitation in Section 3.5.

### 3.3. Cyclic Durability and Fouling Behavior

#### 3.3.1. Changes in Performance over Time

The operating conditions that individually maximized EF and SC were similar but not identical. Within the factorial range, the EF model identified a favorable region near 1.46 V, pH 4.29, and 500 s, whereas the SC model indicated a high-selectivity region near 1.39 V, pH 4.59, and 500 s. Simultaneous numerical optimization produced a compromise condition of approximately 1.43 V, pH 4.48, and 500 s, with the flow rate fixed at 10 mL/min.

At the selected operating condition, analysis of the existing CCD data using the reduced models predicted EF = 2.59 and SC = 3.36. The corresponding 95% confidence intervals for the mean responses were 2.497–2.679 and 3.185–3.540, respectively, while the 95% prediction intervals for an individual observation were 2.374–2.802 and 2.945–3.780 (Supplementary Section S2, Table S3). These intervals are conditional on the retained model structure and error assumptions and do not represent confidence intervals for the optimum operating conditions. Uncertainty in the location of the optimum was estimated separately by a residual bootstrap of the CCD data (Supplementary Section S4, Tables S5 and S6), which gave approximate 95% intervals of 1.41–1.44 V and pH 4.35–4.65, with the sorption time at the 500 s bound in all resamples.

The selected condition was assessed over 10 adsorption/desorption cycles. The reported initial experimental values were EF = 2.54 and SC = 4.71, corresponding to deviations of −1.86% and +40.09% from the model predictions, respectively. The measured EF fell within its 95% prediction interval, whereas SC exceeded the upper prediction limit, so the models reproduce EF quantitatively but not SC.

A possible cause is a difference in component condition between the two stages. The same membrane and electrode set was used for all 20 CCD runs, whereas new components were used for the validation experiments. If deposits accumulated on the CCD component set during the design, as they did on the validation set during the subsequent cycling test, selectivity would be expected to decline more than enrichment, since SC declined by 53.9% and EF by only 15.4% over 100 cycles. Such an offset would displace SC substantially and EF only slightly, which is the pattern observed. However, the CCD component set was not characterized, so this explanation remains a hypothesis; it also implies that absolute optima identified with one component set may not transfer to fresh components. Accordingly, the SC model should be treated as a preliminary guide to operating trends and favorable conditions within the investigated range. Its ability to predict absolute selectivity for different membrane and electrode sets remains unverified.

Performance stability was evaluated over 100 adsorption/desorption cycles under the optimized conditions of 1.43 V, pH 4.48, and 500 s. The test corresponds to approximately 28 h of continuous operation and was designed to resolve the onset and relative kinetics of performance deterioration rather than to qualify the system for extended service. As shown in Figure 7, EF and SC exhibited markedly different rates of deterioration. The enrichment factor remained close to its initial value during the early stage of operation and then gradually decreased from 2.54 to 2.15 by the 100th cycle. This corresponds to a reduction of approximately 15.4%. Although enrichment performance declined, the system retained most of its initial capacity to produce a lithium-enriched concentrate. Selectivity deteriorated more rapidly. SC decreased from 4.71 to 2.17, corresponding to a reduction of approximately 53.9%. Much of this decline occurred during the first 50–70 cycles, after which SC approached a lower and more stable range. The substantially greater decrease in SC than in EF indicates that the ability of the system to discriminate between Li^+^ and divalent transition metals was more vulnerable to repeated cycling than its overall concentration performance.

This difference suggests possible mechanisms of performance deterioration. Partial blockage of electrode pores or flow channels may reduce total electrosorption [53] and thereby lower EF. In contrast, deposition on the MS-CEM or along the cation-transport pathway could alter membrane surface properties [54] and increase the co-transport of Ni^2+^, Co^2+^, and Mn^2+^, which would cause a large loss of SC. The SEM–EDS results presented in the following section are consistent with the association between performance decline and accumulation of transition-metal-containing deposits.

#### 3.3.2. SEM Analysis of Used Module

After the 100-cycle experiment, the module was disassembled and the spacer, electrodes, and ion-exchange membranes were examined using SEM–EDS. The images in Figure 8 and Figure 9 show clear differences between the unused and operated components. As shown in Figure 8a,b, deposits were observed within the spacer openings after 100 cycles operation. EDS analysis of the examined spacer region detected Ni, Co, and Mn at approximately 2.8, 0.6, and 1.8 wt%, respectively. Accumulation within the spacer can reduce the effective flow area, create non-uniform flow paths, and increase concentration polarization [55]. These changes can decrease contact between the feed and active membrane/electrode area and may contribute to the gradual loss of EF.

The electrodes exhibited a strongly asymmetric distribution of deposits. In Figure 8d, the cathode showed extensive surface coverage and contained approximately 11.5 wt% Ni, 6.2 wt% Co, and 12.3 wt% Mn in the analyzed region. In comparison, the anode contained much lower amounts of these metals: approximately 3.5 wt% Ni, 1.1 wt% Co, and 1.1 wt% Mn, as shown in Figure 8e. The preferential accumulation on the cathode is consistent with the migration of positively charged metal ions toward the negatively polarized electrode during adsorption. Local alkalinization at the cathode surface, which was not measured, could additionally favor precipitation of transition-metal hydroxides at this location; this possibility remains a hypothesis. The deposits may cover the external electrode surface and obstruct access to activated-carbon pores, thereby reducing the effective electrosorption capacity.

In Figure 9a,b, the MS-CEM exhibited the most pronounced metal accumulation among the membrane samples. The analyzed MS-CEM surface contained approximately 21.5 wt% Ni, 6.5 wt% Co, and 9.3 wt% Mn after 100-cycle operation. In contrast, the AEM contained substantially smaller amounts of Ni, Co, and Mn, corresponding to approximately 2.3, 0.7, and 1.9 wt%, respectively (Figure 9c,d). This distribution is consistent with preferential deposition along the cation-transport pathway, particularly at the CEM and cathode [56]. Oxygen and sulfur were detected together with the transition metals. These signals are consistent with (hydr)oxide- and/or sulfate-containing deposits, but they cannot be assigned to the deposits alone: the sulfonated cation-exchange membrane contains both elements, the AEM in contact with the sulfate feed holds sulfate as counter-ion, the PET spacer and carbon electrodes contain oxygen, and residual feed salts may contribute to both. Because EDS provides elemental information only, and because no crystallographic (XRD) or chemical-state (XPS) analysis was performed, the phase composition, crystallinity and thickness of the deposits were not determined and the proposed composition remains a hypothesis.

The SEM–EDS observations are consistent with, but do not by themselves establish, the following interpretation of the EF and SC deterioration. Deposition on the electrode and spacer could reduce the overall ion-storage and transport capacity, whereas extensive coverage of the MS-CEM could additionally interfere with the monovalent-selective transport mechanism. The greater sensitivity of SC is therefore consistent with the particularly heavy transition-metal accumulation observed on the MS-CEM. Accordingly, chemical cleaning was evaluated to determine whether the performance loss was reversible.

Table 8 separates the conclusions that follow directly from the measurements from those that remain proposed explanations. The single-point nature of the EDS analysis and the absence of membrane transport measurements before and after fouling mean that the mechanism by which deposition reduces selectivity is inferred from the performance data and the elemental distribution rather than measured directly [57,58].

#### 3.3.3. Application of Cleaning

The effectiveness of the sequential EDTA and HCl cleaning procedure was evaluated by comparing EF and SC cycles before cycling, after 100 cycles, and after cleaning. The results are presented in Figure 10. After 100 cycles, EF had decreased from 2.54 to 2.15. Cleaning increased EF to 2.25, corresponding to approximately 89% of the initial value. Thus, cleaning produced only a moderate recovery of enrichment performance. The incomplete recovery suggests that part of the performance loss may have resulted from residual pore blockage, incomplete deposit removal, or partially irreversible changes in the electrode structure.

The recovery of selectivity was substantially greater. SC increased from 2.17 after 100 cycles to 4.38 after cleaning, corresponding to approximately 93% of its initial value of 4.71. Expressed as a fraction of the performance lost during cycling, cleaning recovered ap-proximately 87% of the SC loss (2.21 of 2.54) but only 26% of the EF loss (0.10 of 0.39). The near-restoration of SC suggests that a large fraction of the selectivity loss was reversible and may have been associated with removable deposits on the membrane and other cation-transport surfaces. EDTA can chelate accumulated transition metals, whereas the subsequent HCl treatment can dissolve residual inorganic deposits [59]. The different recovery behaviors of EF and SC are consistent with the interpretation that membrane-surface fouling was a major contributor to the loss of selectivity, while electrode-related limitations contributed more strongly to the persistent reduction in enrichment performance.

These findings show that a single EDTA/HCl cleaning sequence substantially restored the lithium-selective performance of the MCDI module; whether this recovery can be maintained over repeated fouling–cleaning cycles was not tested. For practical application, however, cleaning frequency, chemical consumption, cleaning-solution disposal, lithium loss during cleaning, and long-term membrane and electrode durability should be evaluated. Optimization of the cleaning protocol will therefore be necessary to balance sustained selectivity against operational cost and secondary waste generation.

Only a single fouling–cleaning sequence was evaluated in this study, and the recovery observed after one cleaning cannot be extrapolated to repeated cleaning during extended operation. Two mechanisms are relevant. First, the monovalent selectivity of commercial MS-CEMs originates from a thin, positively charged surface layer applied to a conventional cation-exchange membrane, and this layer is thinner and mechanically weaker than the bulk membrane [45,49]. Because the selectivity of the present system is expected to depend largely on that layer, even limited erosion, partial charge neutralization, or the swelling and deswelling stress associated with repeated ionic-form conversion would reduce SC disproportionately relative to EF. Reported consequences of repeated chemical cleaning of ion-exchange membranes include partial loss of fixed functional groups, formation of surface and bulk microcavities that subsequently act as foulant traps, increased area resistance and reduced permselectivity; in some cases, the damage caused by aggressive cleaning is comparable to that caused by the fouling itself [57,59,60]. Second, on the electrode side, repeated acid exposure could promote oxidation of carbon surface functional groups and weaken the binder, and EDTA could be retained within the microporous structure; both effects, which were not examined here, would reduce the effective electrosorption capacity. The incomplete recovery of EF observed after a single cleaning (26% of the EF loss recovered, compared with 87% of the SC loss) is consistent with a partially irreversible electrode-side contribution, which could also include anodic oxidation of the carbon during cycling [51].

The concentrations used here (0.01 M EDTA and 0.01 M HCl) are lower than those commonly used for cleaning-in-place, which may reduce, but does not eliminate, this risk. Establishing a cleaning frequency suitable for continuous operation would require repeated fouling–cleaning sequences accompanied by measurement of area resistance, ion-exchange capacity, permselectivity, water contact angle and electrode capacitance before and after each cleaning. Such measurements were not performed in the present work and are identified as the principal requirement for future study (Section 3.5).

### 3.4. Process Performance Indicators and Comparison with Alternative Technologies

The enrichment factor and the selectivity coefficient describe the composition of the concentrate relative to the feed, but they do not by themselves establish the practical performance of the process. The performance indicators that could be derived from the present data set are summarized in Table 9. Over the 100-cycle test, EF reached a maximum of 2.54 and decreased to 2.15, while SC decreased from 4.71 to 2.17, indicating deterioration in lithium enrichment and, in particular, in selectivity. Based on ICP-AES analysis of Li^+^ in the treated stream, Li^+^ removal during adsorption was 83–92%. The Li^+^ molar fraction among the four metals in the concentrate decreased from 90.2% to 81.0%, compared with 66.7% in the feed. The concentrate volume corresponded to 36% of the feed volume treated in each 10-cycle run (300 mL per 833 mL), giving a volumetric productivity of 1.35 L m^−2^ h^−1^ based on the effective membrane area of 0.080 m^2^ (eight MS-CEMs of 100 × 100 mm). Metal-ion charge efficiency, calculated with Equation (5), was 7.6–8.7%, indicating that only a small fraction of the measured charge was associated with the quantified net removal of metal cations. Possible contributions include faradaic reactions, incomplete charge utilization, and electrical leakage; however, these contributions were not measured separately and cannot be ranked from the present data.

Lithium recovery was determined from the lithium balance over each 10-cycle run. The lithium input was therefore 31.3 mg per run (37.6 mg/L × 10 × 83.3 mL), or 313 mg over 100 cycles. The lithium accumulated in the loop, obtained from the measured loop concentration and volume, was 23.9–28.8 mg per run. This corresponds to lithium recoveries of 76.2–91.8%, with the remainder leaving in the treated stream. Recovery was close to the removal efficiency during adsorption, indicating that most of the lithium removed from the feed was transferred to the concentrate. In contrast to lithium, the fraction of the divalent metals transferred to the concentrate increased from about 16% to 36% over the test, which accounts for the decline in SC and in the Li^+^ molar fraction of the concentrate.

The specific energy consumption estimated with Equation (6) ranged from 0.201 to 0.324 kWh/mol Li^+^, where the lithium collected is the lithium accumulated in the concentrate loop. This range partially overlaps the reported electrodialysis range of 0.04–0.27 kWh/mol Li^+^ [61,62], although the upper value is approximately 20% higher. The present estimates are therefore comparable in magnitude to the upper end of these reported values. Nevertheless, they remain substantially higher than the 0.0018 kWh/mol Li^+^ reported for MS-CEM-assisted MCDI separating Li^+^ from Mg^2+^. Differences in feed composition, separation objectives, and energy-accounting methods limit direct comparison. The relatively high estimates may partly reflect operating conditions optimized for enrichment and selectivity without including energy consumption as an objective. Possible resistive and Faradaic losses, together with the leakage currents discussed above, could also contribute, although their contributions were not quantified.

A comparison with the conventional alternatives for lithium recovery from dilute streams is presented in Table 10. Conventional precipitation can remove divalent metals but may cause lithium co-precipitation losses of up to 30% [63]. An integrated solvent-extraction and precipitation process reported > 90% extraction of Co, Mn, and Ni and a final Li_2_CO_3_ purity exceeding 99% [64]. Lithium-ion sieves maintained recovery above 80% over five adsorption–desorption cycles [65], while selective electrodialysis achieved a Li^+^/Co^2+^ permselectivity of 17.3 [66]. For comparison, MS-CEM-assisted MCDI has been reported to provide a Li^+^/Mg^2+^ selectivity coefficient of 2.95 [20]. In the present study, EF = 2.54 and SC = 4.71 were obtained using a synthetic feed containing approximately 37.6 mg/L Li^+^ and three competing divalent metals. Differences in feed composition, selectivity definitions, and recovery objectives prevent direct ranking of these technologies. The present results indicate the potential of MS-CEM-assisted MCDI for selective lithium preconcentration before downstream purification. However, EF and SC alone do not establish advantages in lithium mass recovery or chemical consumption, and additional purification remains necessary to obtain a high-purity lithium product.

### 3.5. Limitations of This Study

Several limitations should be considered when interpreting these results. The experiments used synthetic LIB-manufacturing wastewater with a fixed ion ratio under mildly acidic conditions and at room temperature. Real wastewater may contain variable metal concentrations, background salts, organic matter, and suspended solids that alter ion competition and fouling. Accordingly, the condition of 1.43 V, pH 4.48, and 500 s represents a local optimum for the investigated feed and operating range rather than a universally applicable condition.

The reduced quadratic models were statistically significant, and their lack-of-fit tests were not significant at the 5% level (*p* = 0.0822 for EF and *p* = 0.0776 for SC). However, both probabilities were close to 0.05, and the experimentally observed SC at the selected condition (4.71) exceeded both the value predicted by the model at that condition (3.36) and the highest value measured anywhere within the central composite design (3.56). The validation and cycling experiments were performed on a newly installed membrane and electrode set, whereas the central composite design was executed as a continuous block on a single component set whose condition was not characterized. Progressive metal accumulation during the design, analogous to that observed in the subsequent cycling test, may explain why the selectivity measured with new components exceeded the model prediction, but this explanation was not verified. The response surface models should accordingly be interpreted as describing the relative influence of the operating factors and the location of the favorable operating region, rather than as predictors of absolute performance for an arbitrary component set. Independent validation at additional factor combinations is therefore required before extrapolation or scale-up. A sequential higher-order design may be warranted if systematic curvature persists [53].

EF and SC describe lithium concentration and relative separation from divalent metals but do not establish lithium mass recovery, product purity, or overall process efficiency. Future studies should close the lithium mass balance and report recovery, purity, and energy consumption per unit mass of lithium recovered. Testing with real wastewater, longer operation, and repeated fouling-cleaning cycles is also required to determine process durability and cleaning demand. Until these aspects are verified, the present MCDI system is best considered a selective pretreatment or pre-concentration step before downstream hydrometallurgical recovery.

Several further limitations should be stated explicitly. A faradaic contribution at the higher voltages investigated may have contributed to the residual variance of the response models. Charge efficiency was estimated only at the cyclic operation condition, and coulombic efficiency, effluent pH and gas evolution were not measured, so the extent of faradaic re-actions was not quantified. Deposit characterization was limited to SEM and EDS, which provide elemental composition without chemical-state or crystallographic information, so the mineralogical identity, crystallinity, particle morphology and thickness of the deposits were not determined. EDS was performed at a single location per component, so comparisons between components are not statistically representative. Membrane and electrode transport properties, including area resistance, ion-exchange capacity, permselectivity, contact angle and specific capacitance, were not measured before and after fouling, so the mechanism by which deposition reduces selectivity is inferred rather than measured. Only a single fouling–cleaning sequence was evaluated, and the durability of the cleaning protocol over repeated application was not assessed. Physical characterization of the activated-carbon electrodes was not available, so the reported performance is specific to the electrodes used. Finally, the pH of the feed was adjusted with H_2_SO_4_, and sulfate forms neutral ion pairs with the divalent metals under the conditions investigated; the reported selectivity coefficients may therefore reflect the combined effect of membrane monovalent selectivity and solution-phase speciation, and quantitative speciation calculations were not performed. The acid volumes were not recorded, so the added sulfate is known only as a lower bound. The Li^+^/Ni^2+^ selectivity coefficient remained the lowest of the three ion pairs throughout, so downstream purification, for example by selective precipitation of Ni, Co and Mn, solvent extraction or chelating ion exchange, would still be required before lithium could be recovered as a battery-grade product.

The 100-cycle test corresponds to approximately 28 h of continuous operation. This duration was sufficient to distinguish the slow decline of EF from the substantially faster decline of SC and to capture the stabilization of SC after 50–70 cycles, which was the objective of the experiment, but it is not sufficient to establish membrane or electrode lifetime. The mechanical and dimensional integrity of the membranes after cycling was not assessed. Durability assessment for practical pretreatment would require operation over several hundred to several thousand cycles with periodic cleaning, together with post-operation evaluation of membrane integrity, including dimensional stability, burst strength, ion-exchange capacity and area resistance.

## 4. Conclusions

This study evaluated MS-CEM-assisted MCDI for selective lithium enrichment from simulated LIB-manufacturing wastewater. OFAT experiments identified sorption time as an influential factor and revealed a trade-off between enrichment and selectivity when the adsorption/desorption time ratio was varied. RSM identified a favorable local operating region and a significant pH–sorption time interaction for SC. At 1.43 V, pH 4.48, 500 s, 10 mL/min, and a 1:1 time ratio, the initial EF and SC were 2.54 and 4.71. The measured EF agreed with the predicted value of 2.59, whereas SC exceeded the prediction of 3.36 and its prediction interval. The models therefore support interpretation of local operating trends, while quantitative prediction of SC remains limited.

Over 100 cycles, corresponding to approximately 28 h, EF and SC declined by 15.4% and 53.9%, respectively. Selectivity was therefore more sensitive to repeated operation than lithium enrichment. SEM–EDS detected transition metals on all examined components, with the highest contents at the analyzed locations on the cathode and MS-CEM, consistent with an association between deposition and performance deterioration. Deposit phases and changes in membrane transport properties were not determined. A single sequential cleaning with 0.01 M EDTA and 0.01 M HCl increased EF to 2.25 and SC to 4.38, corresponding to approximately 89% and 93% of their initial values. The greater restoration of selectivity suggests a substantial reversible contribution to its decline, although repeated-cleaning durability was not established.

The nominal concentrate volume fraction was 36%, with a productivity of 1.35 L m^−2^ h^−1^ for a membrane area of 0.080 m^2^. The calculated net specific energy consumption was 0.201–0.324 kWh/mol Li^+^. These estimates depend on the stated removal, collection-volume, and energy-accounting assumptions. The comparatively low Li^+^/Ni^2+^ selectivity indicates that improved nickel discrimination and downstream purification are necessary. The present results support the need for selective pre-concentration before conventional Li^+^ recovery. Testing with real wastewater, extended cycling, repeated cleaning, and verified recovery and energy measurements are required to assess practical applicability.

## Figures and Tables

**Figure 1:**
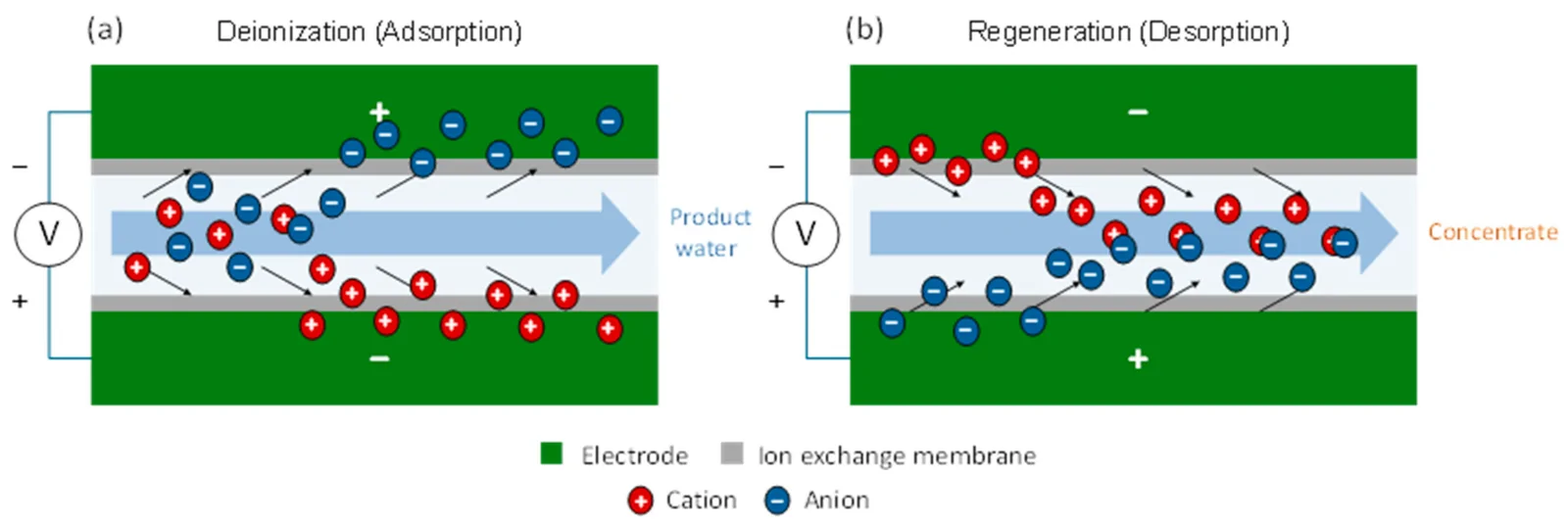
Schematic structure of the MCDI system: (**a**) Adsorption time; (**b**) Desorption time.

**Figure 2:**
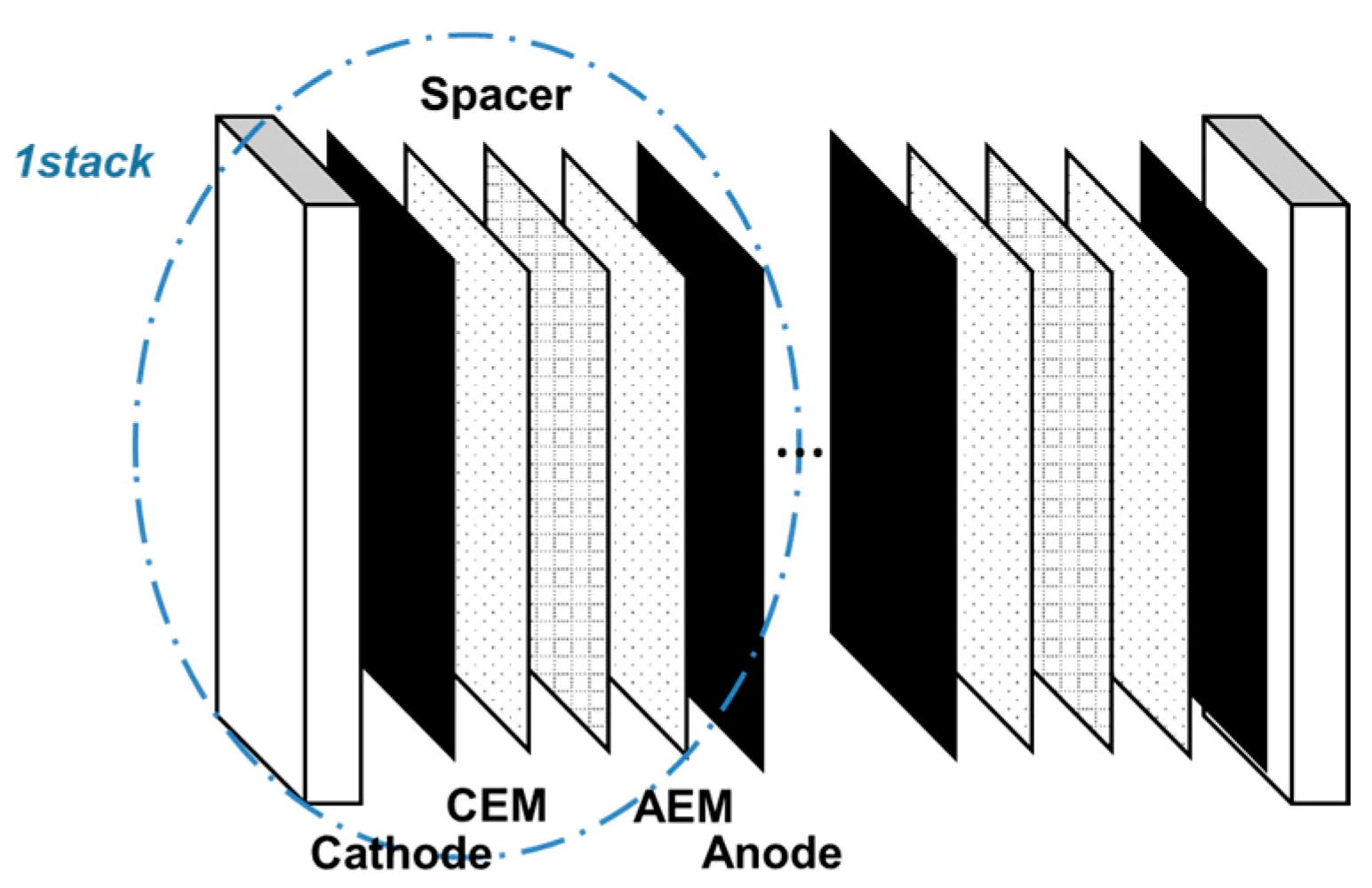
Schematic diagram of the 8-stack MCDI module.

**Figure 3:**
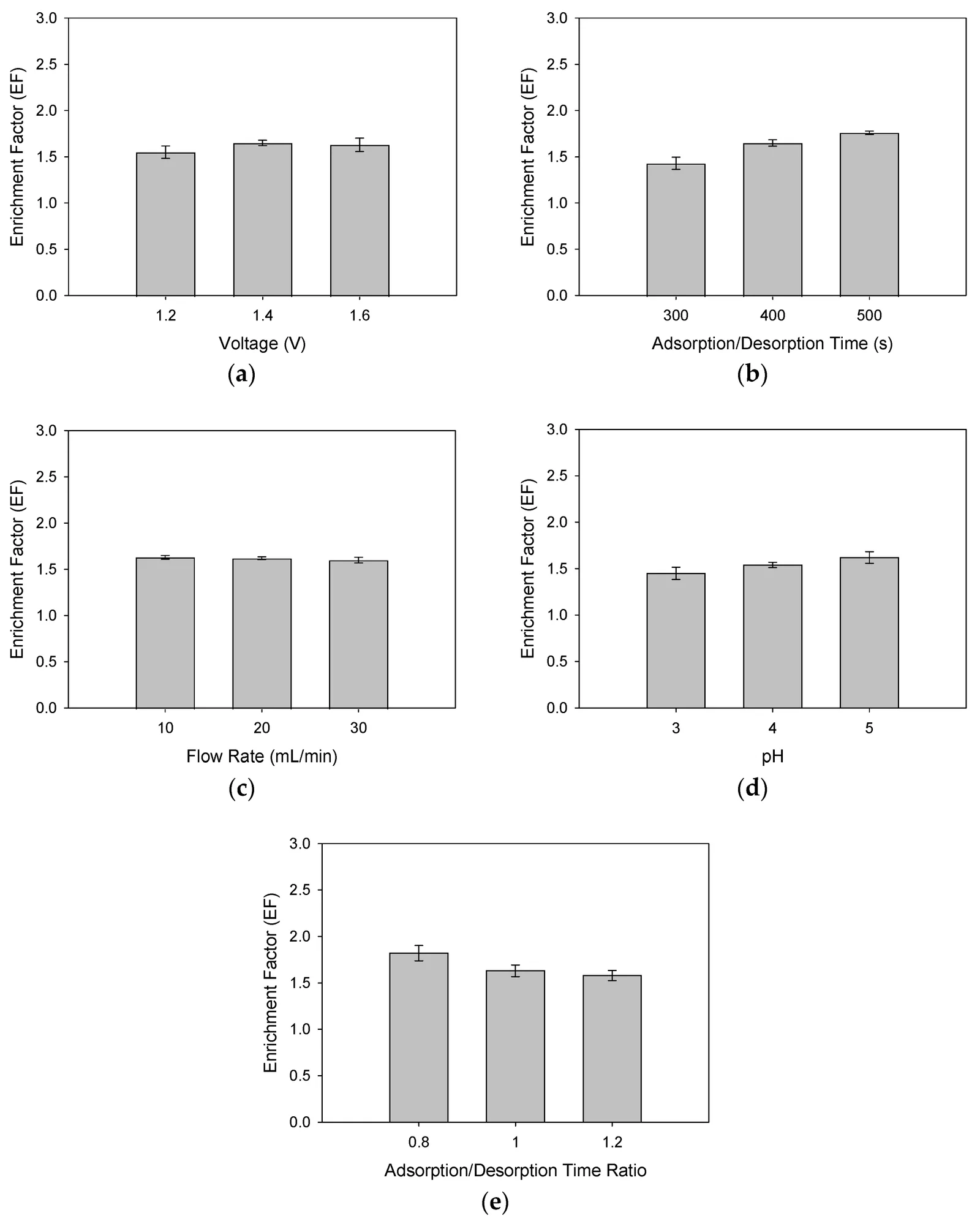
Lithium enrichment factor obtained from OFAT experiments evaluating the effects of (**a**) applied voltage, (**b**) adsorption/desorption time, (**c**) flow rate, (**d**) feed pH, and (**e**) adsorption/desorption time ratio. Error bars represent standard deviations (*n* = 3).

**Figure 4:**
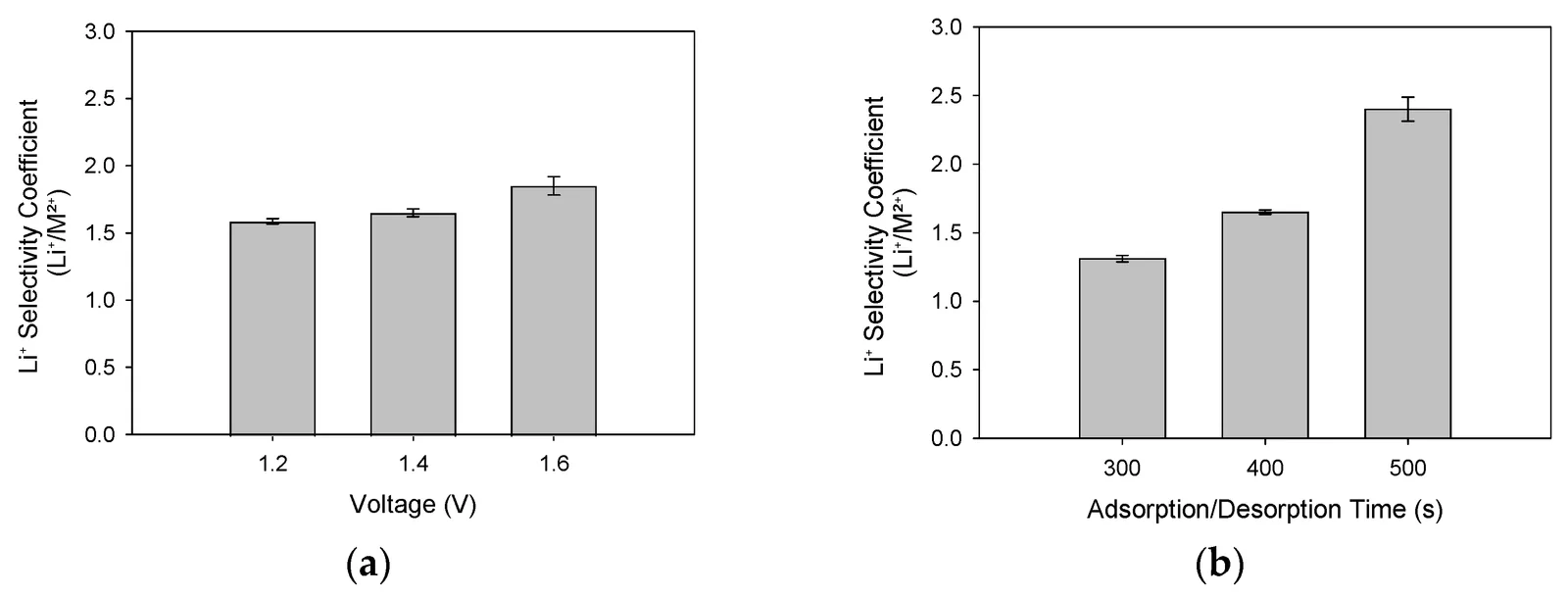

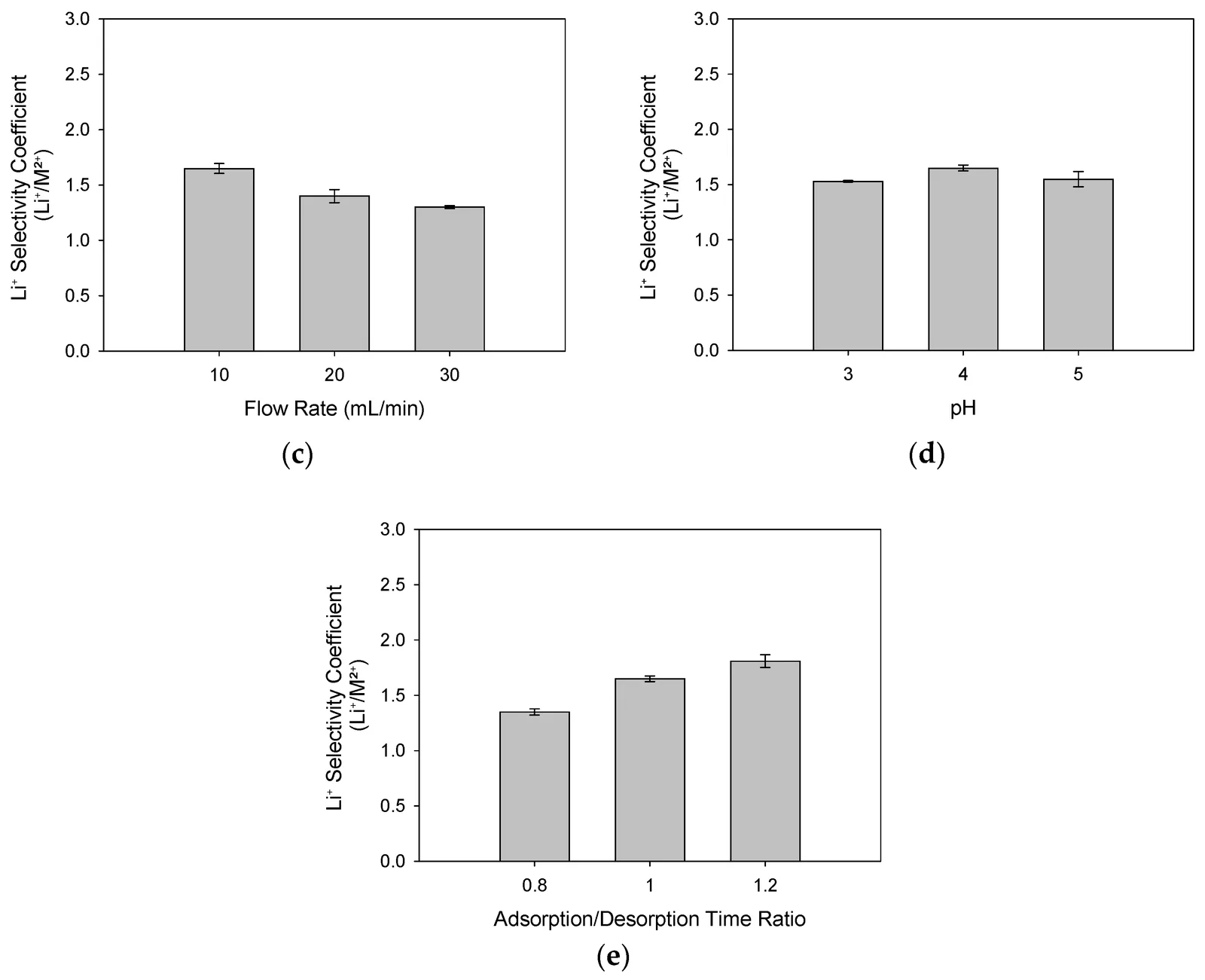
Average Li^+^/M^2+^ selectivity coefficient obtained from OFAT experiments evaluating the effects of (**a**) applied voltage, (**b**) adsorption/desorption time, (**c**) flow rate, (**d**) feed pH, and (**e**) adsorption/desorption time ratio. Error bars represent standard deviations (*n* = 3).

**Figure 5:**
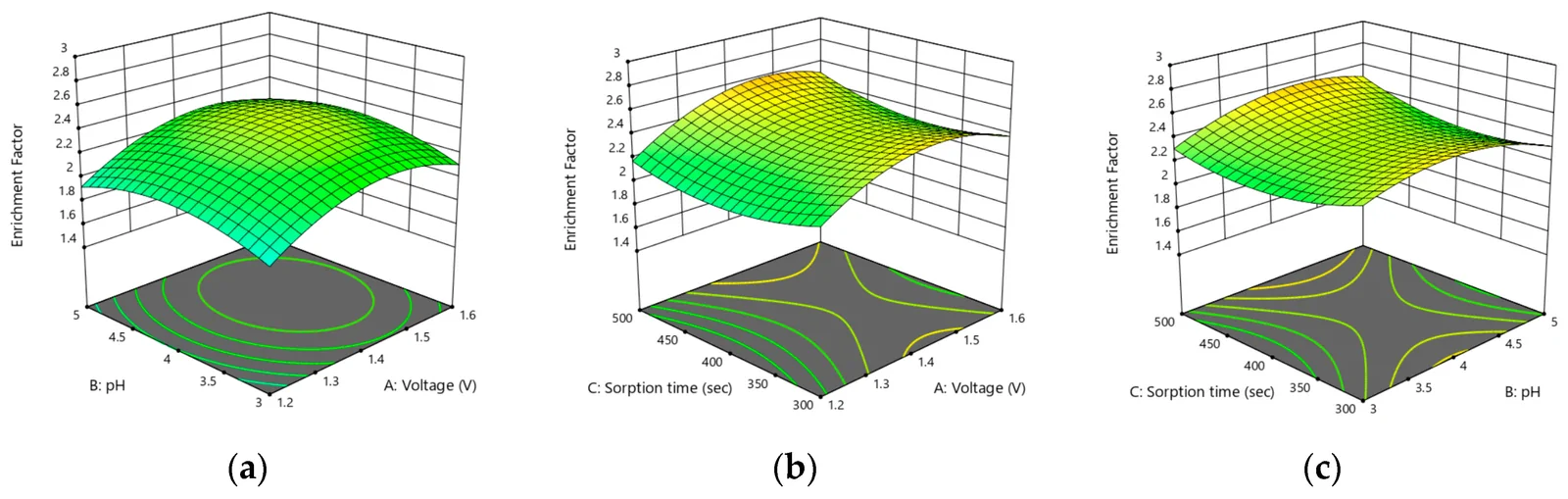
Response surface plots showing the effects of (**a**) voltage and pH, (**b**) voltage and sorption time, and (**c**) pH and sorption time on the lithium enrichment factor.

**Figure 6:**
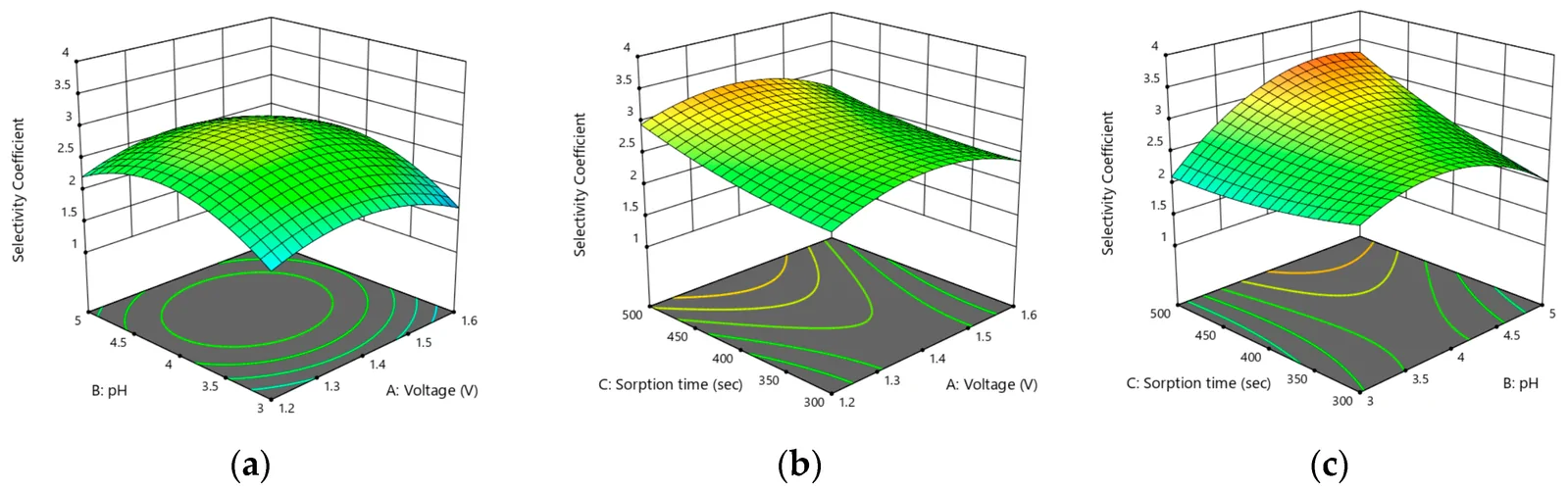
Response surface plots showing the effects of (**a**) voltage and pH, (**b**) voltage and sorption time, and (**c**) pH and sorption time on the Li^+^/M^2+^ selectivity coefficient.

**Figure 7:**
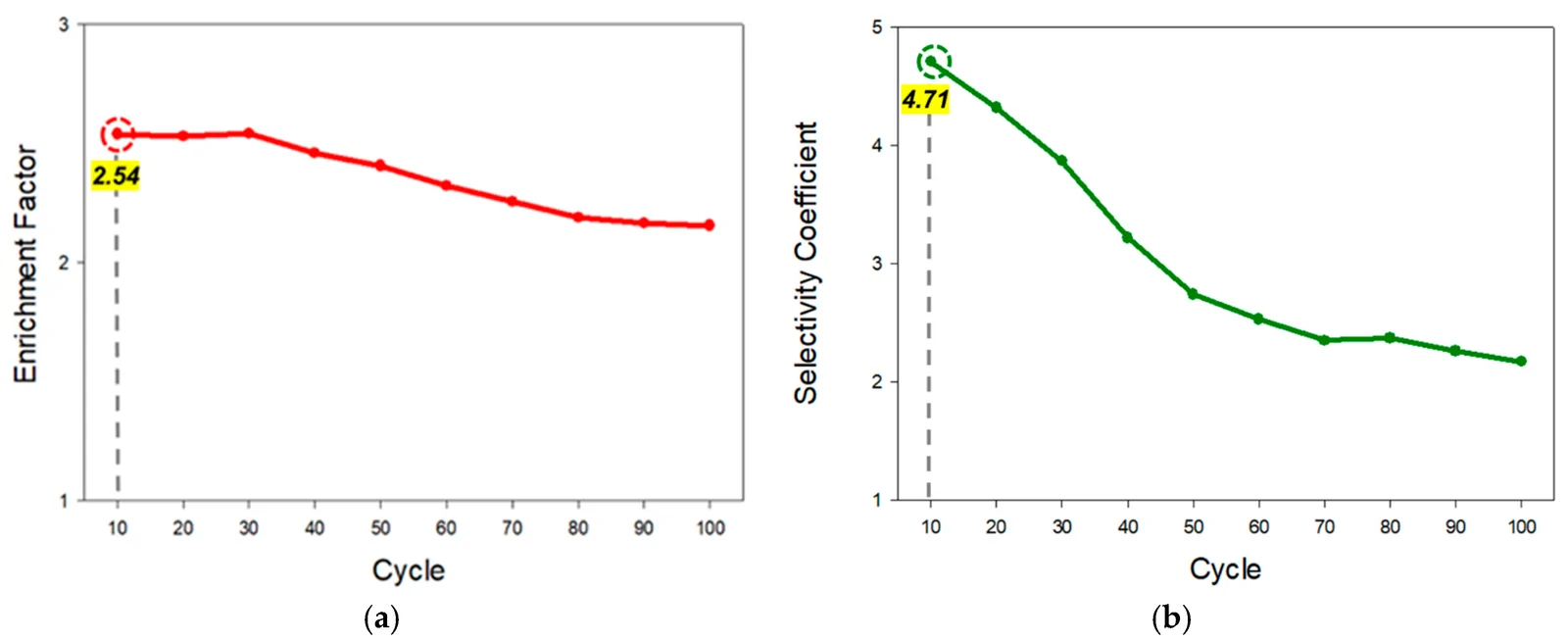
Operation performance of the MCDI system over 100 cycles, showing changes in (**a**) enrichment factor and (**b**) Li^+^/M^2+^ selectivity coefficient.

**Figure 8:**
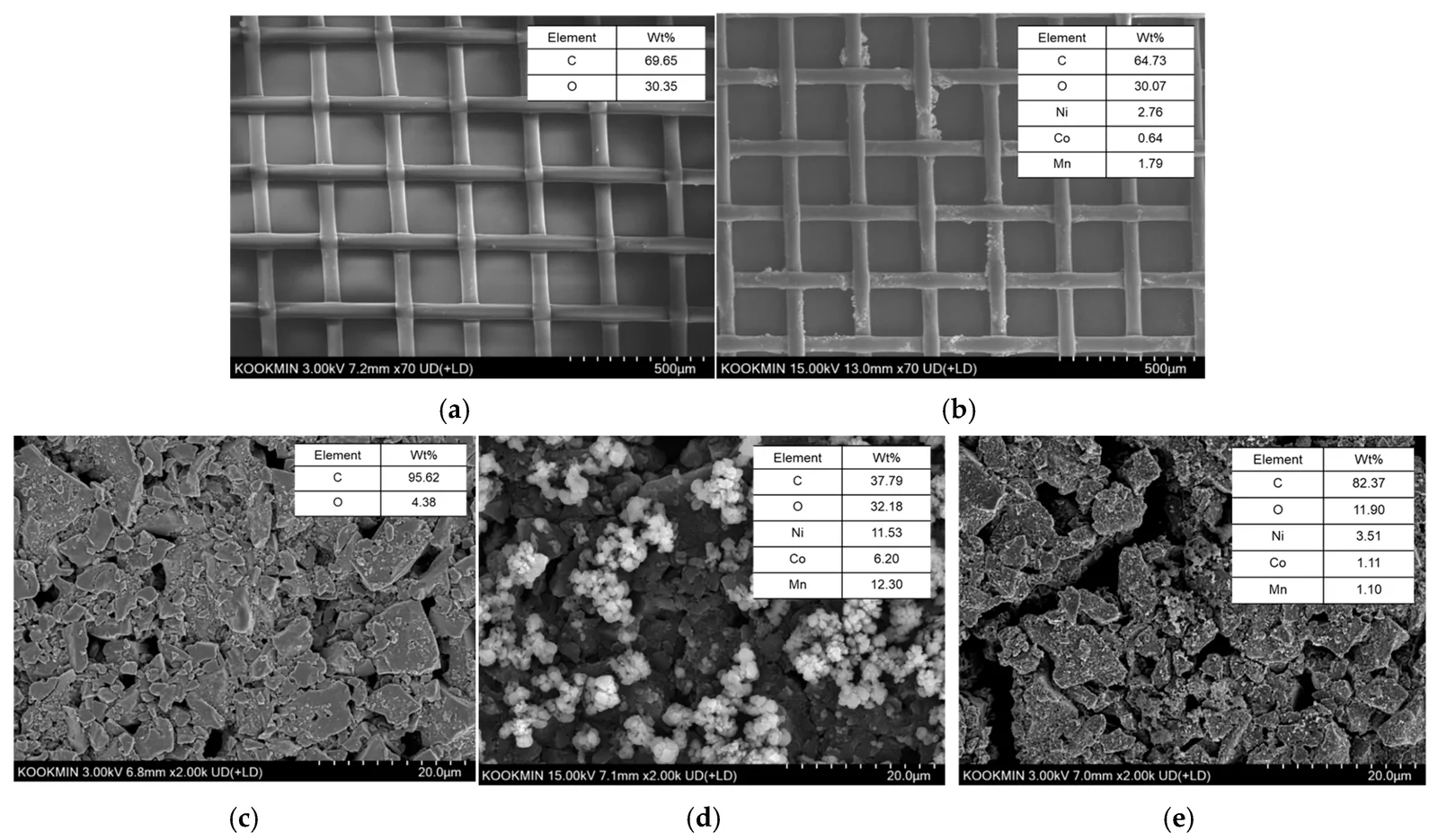
FE-SEM images and EDS results for the spacer and electrodes before and after 100-cycle operation: (**a**) spacer before operation, (**b**) spacer after operation, (**c**) electrode before operation, (**d**) cathode after operation, and (**e**) anode after operation.

**Figure 9:**
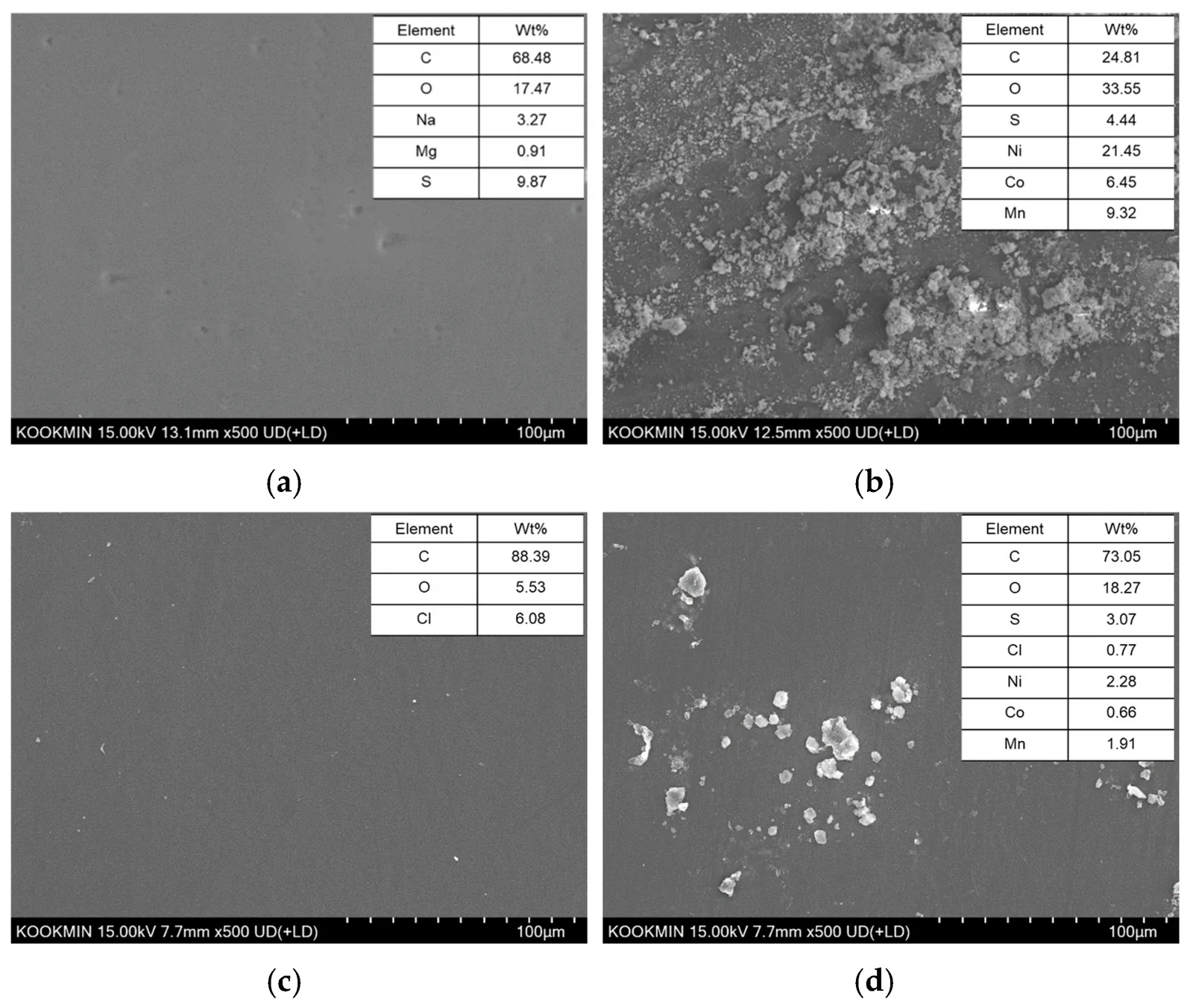
FE-SEM images and EDS results for the ion-exchange membranes before and after 100-cycle operation: (**a**) MS-CEM before operation, (**b**) MS-CEM after operation, (**c**) AEM before operation, and (**d**) AEM after operation.

**Figure 10:**
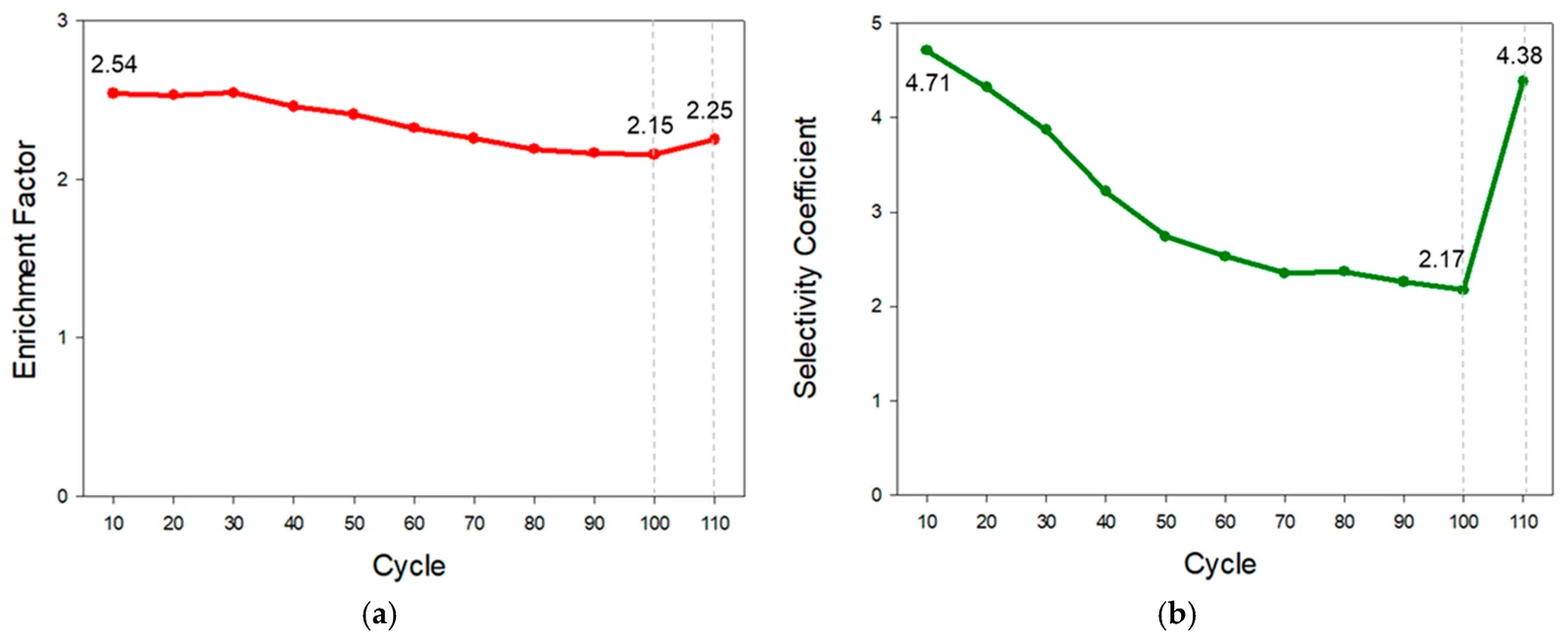
Recovery of (**a**) enrichment factor and (**b**) Li^+^/M^2+^ selectivity coefficient following sequential EDTA and HCl cleaning.

**Table 1:** Compositions of the feed solution.

Composition	Concentration (mg/L)
LiCl	229.6
NiSO_4_·6H_2_O	426.9
CoSO_4_·7H_2_O	152.2
MnSO_4_·H_2_O	91.5

**Table 2:** Summary of components in MCDI module.

Component	Manufacturer	Material	Dimensions (mm)
Electrode	PureeChem, Cheongju, Republic of Korea	Activated carbon, Graphite	100 × 100 × 0.6
Spacer	PureeChem, Cheongju, Republic of Korea	Woven polyethylene ter-ephthalate mesh	110 × 110 × 0.1
MS-CEM	ASTOM Corporation, Tokyo, Japan	Styrene-Divinylbenzene with cation-selective surface layer	100 × 100 × 0.15
Standard CEM	ASTOM Corporation, Tokyo, Japan	Styrene-Divinylbenzene (reinforced)	100 × 100 × 0.17
Standard AEM	ASTOM Corporation, Tokyo, Japan	Styrene-Divinylbenzene (reinforced)	100 × 100 × 0.14

**Table 3:** Factors and responses used in RSM.

Type	Data	Unit	Value
Factor	Voltage	V	1.06, 1.2, 1.4, 1.6, 1.74
	pH	-	2.32, 3, 4, 5, 5.68
	Sorption time	s	232, 300, 400, 500, 568
Response	Enrichment factor	-	
	Selectivity coefficient	-	

The symbol (-) indicates that pH has no unit.

**Table 4:** Li^+^/multivalent ion selectivity under different operating variables in OFAT experiments.

Variable	Condition	Li^+^/Ni^2+^	Li^+^/Co^2+^	Li^+^/Mn^2+^	Li^+^/M^2+^ Avg.
Voltage (V)	1.2	1.12	1.57	2.56	1.59
	1.4	1.25	1.73	2.17	1.65
	1.6	1.34	1.94	2.62	1.85
Adsorption/Desorption time (s)	300	1.09	1.38	1.56	1.31
	400	1.25	1.75	2.17	1.65
	500	1.82	2.54	3.24	2.4
Flow rate (mL/min)	10	1.52	1.68	1.77	1.65
	20	1.33	1.41	1.47	1.4
	30	1.27	1.30	1.33	1.3
pH	3	1.14	2.07	1.66	1.53
	4	1.26	1.75	2.18	1.65
	5	1.1	2.34	1.68	1.55
Adsorption/Desorption time ratio	1:0.8	1.08	1.45	1.67	1.35
	1:1	1.26	1.75	2.18	1.65
	1:1.2	1.21	2.06	2.91	1.81

**Table 5:** RSM results on enrichment factor (EF) and selectivity coefficient (SC).

Run	Factor 1 A: Voltage (V)	Factor 2 B: pH	Factor 3 C: Sorption Time	Response 1 Enrichment Factor	Response 2 Selectivity Coefficient
1	1.2	5	500	2.15	3.06
2	1.06364	4	400	1.44	2.07
3	1.6	3	300	2.18	1.85
4	1.4	4	400	2.35	2.99
5	1.4	4	568.179	2.86	3.56
6	1.4	4	400	2.45	2.75
7	1.4	4	400	2.41	2.94
8	1.4	4	400	2.48	2.78
9	1.6	5	300	2.17	1.67
10	1.2	3	500	1.95	1.77
11	1.4	4	231.821	2.73	3.03
12	1.6	5	500	2.39	2.87
13	1.6	3	500	2.14	1.54
14	1.4	5.68179	400	1.97	1.74
15	1.4	4	400	2.35	2.95
16	1.2	5	300	2.09	1.53
17	1.73636	4	400	2.12	1.84
18	1.4	2.31821	400	1.96	1.39
19	1.4	4	400	2.45	2.78
20	1.2	3	300	2.12	1.94

**Table 6:** ANOVA for the response surface quadratic model: Enrichment Factor (EF).

Source	Sum of Squares	df	Mean Square	F-Value	*p*-Value
Model	1.7	7	0.2435	30.82	<0.0001
A-Voltage	0.215	1	0.215	27.22	0.0002
B-pH	0.0133	1	0.0133	1.69	0.2182
C-Sorption time	0.0061	1	0.0061	0.7723	0.3968
BC	0.03	1	0.03	3.8	0.075
A^2^	0.7498	1	0.7498	94.92	<0.0001
B^2^	0.3814	1	0.3814	48.29	<0.0001
C^2^	0.2464	1	0.2464	31.2	0.0001
Residual	0.0948	12	0.0079		
Lack of Fit	0.0796	7	0.0114	3.75	0.0822
Pure Error	0.0152	5	0.003		
Cor Total	1.8	19			

**Table 7:** ANOVA for the response surface quadratic model: Selectivity Coefficient (SC).

Source	Sum of Squares	df	Mean Square	F-Value	*p*-Value
Model	8.1653	7	1.1665	38.81	<0.0001
A-Voltage	0.0419	1	0.0419	1.40	0.2604
B-pH	0.5021	1	0.5021	16.70	0.0015
C-Sorption time	0.7226	1	0.7226	24.04	0.0004
BC	1.2880	1	1.2880	42.85	<0.0001
A^2^	1.9241	1	1.9241	64.01	<0.0001
B^2^	3.6503	1	3.6503	121.44	<0.0001
C^2^	0.1692	1	0.1692	5.63	0.0352
Residual	0.3607	12	0.0301		
Lack of Fit	0.3046	7	0.0435	3.87	0.0776
Pure Error	0.0562	5	0.0112		
Cor Total	8.5260	19			

**Table 8:** Experimental observations obtained in this study and the corresponding level of support for the proposed fouling mechanism.

Directly Measured Observation	Directly Supported Conclusion	Proposed Interpretation (Not Verified in This Study)
Ni, Co and Mn detected by EDS on spacer, electrodes and MS-CEM at the analyzed locations after 100 cycles	Transition metals accumulated on MCDI components during repeated operation	—
Metal content much higher on cathode than anode, and on MS-CEM than AEM	Accumulation is asymmetric and concentrated on the cation-transport side (single-location analyses; not statistically representative of whole com-ponents)	Driven by electromigration of M^2+^ towards the negatively polarized electrode; possibly favored by local alkalinization at the cathode (hypothesis)
EF declined by 15.4% and SC by 53.9% over 100 cycles	The two performance indicators degrade at different rates	Selectivity loss dominated by MS-CEM surface deposition; enrichment loss dominated by electrode and spacer effects; anodic oxidation of the carbon may also contribute
O and S detected together with Ni, Co and Mn	O and S are pre-sent at the analyzed sur-faces; their assignment to the deposits is uncertain because the substrates also contain O (and the membranes S)	Metal (hydr)oxide and/or sulfate phases; not confirmed, as no XRD or XPS data were obtained
SC recovered to 93% and EF to 89% of initial values after EDTA/HCl cleaning (87% of the SC loss and 26% of the EF loss recovered)	A large fraction of the selectivity loss, but only a small fraction of the EF loss, was reversed by the disassembly–cleaning sequence	Reversible fraction corresponds to chelatable and acid-soluble surface deposits

(—) Indicates that no further interpretation is required beyond the directly supported conclusion.

**Table 9:** Process performance indicators at the optimized condition (1.43 V, pH 4.48, 500 s, 10 mL/min, adsorption/desorption time ratio 1:1).

Performance Indicator	Definition	Value at the Cyclic Operation Condition
Li^+^ enrichment factor, EF	Equation (2)	2.15–2.54
Li^+^/M^2+^ selectivity coefficient, SC	Equation (4)	2.17–4.71
Li^+^ removal efficiency during adsorption	Equation (1)	83–92%
Li^+^ molar fraction of the concentrate	*n*_Li_^+^/[*n*_Li_^+^ + *n*_Ni_^2+^ + *n*_Co_^2+^ + *n*_Mn_^2+^]	81.0–90.2%
Li^+^ recovery	Mass of Li^+^ collected in concentrate/Total mass of Li^+^ introduced during the cycle	76.2–91.8%
Concentrate fraction (water recovery per 10 cycles)	Concentrate volume/feed volume per 10 cycles	36%
Volumetric productivity	Concentrate volume per unit membrane area per hour	1.35 L m^−2^ h^−1^ if A_m_ = 0.080 m^2^
Charge efficiency	Equation (5)	7.6–8.7%
Specific energy consumption, SEC	Equation (6)	0.201–0.324 kWh/mol Li^+^

**Table 10:** Qualitative comparison of technologies for lithium recovery or pre-concentration from dilute aqueous streams.

Technology	Performance Reported	References
Precipitation (NaOH, Na_2_CO_3_)	Co-precipitation losses of up to 30% of the lithium inventory; leaching transfers > 99% of metals to solution, so the loss arises at the precipitation stage.	[63]
Solvent extraction (D2EHPA, Cyanex 272)	>90% co-extraction of Co, Mn and Ni with Li^+^ left in the raffinate; Li_2_CO_3_ purity > 99%.	[64]
Adsorption/lithium-ion sieves	Desorption efficiency > 90%; recovery >80% retained through five adsorption–desorption cycles.	[65]
Selective electrodialysis	Li^+^/Co^2+^ permselectivity 17.3 and approx. 20 kWh/kg Li on simulated LIB.	[66]
Selective electrodialysis	Li^+^ recovery 77–90%; specific energy 2.6–34 kWh/kg Li depending on current density.	[67]
Selective electrodialysis	Literature range 0.04–0.27 kWh/mol Li for electrodialysis.	[20]
MS-CEM MCDI (literature, Li^+^/Mg^2+^)	SC = 2.95; 0.0018 kWh/mol Li.	[20]
MS-CEM MCDI	EF = 2.54; SC = 4.71; 0.201–0.324 kWh/mol Li^+^.	this study

## Data Availability

The data presented in this study are available from the corresponding author upon reasonable request.

## Supplementary Materials

The following supporting information can be downloaded at: https://www.mdpi.com/article/10.3390/membranes16100324/s1, Table S1, Reconstructed model statistics; Table S2, Coefficients and conditional 95% confidence intervals; Table S3, Conditional predictions and comparison with the reported initial responses; Table S4, Fitted responses and influence measures by Table 5 run label; Table S5, Sensitivity of the model compromise to response importance; Table S6, EF sorption-time comparison at the common reference voltage and pH; Figure S1: EF diagnostics from the existing CCD data. Q–Q and residual plots describe model residuals; they do not establish external predictive validity or rule out unmeasured mechanisms; Figure S2: SC diagnostics from the existing CCD data. Q–Q and residual plots describe model residuals; they do not establish external predictive validity or rule out unmeasured mechanisms. References [68,69,70] are cited in the Supplementary Materials.

## Abbreviations

The following abbreviations are used in this manuscript:

AEM	Anion-exchange membrane
ANOVA	Analysis of variance
CCD	Central composite design
CDI	Capacitive deionization
CEM	Cation-exchange membrane
EDTA	Ethylenediaminetetraacetic acid
EDS	Energy-dispersive X-ray spectroscopy
EF	Enrichment factor
FE-SEM	Field-emission scanning electron microscopy
ICP-AES	Inductively coupled plasma atomic emission spectrometry
LIB	Lithium-ion battery
MCDI	Membrane capacitive deionization
MS-CEM	Monovalent-selective cation-exchange membrane
NCM	Nickel–cobalt–manganese
OFAT	One-factor-at-a-time
RSM	Response surface methodology
SC	Selectivity coefficient
