# Supplementary material for: Performance Analysis and Optimization of a Membrane Capacitive Deionization Process for Lithium Enrichment from Lithium-Ion Battery Wastewater
Source: Membranes (Basel). 2026 Sep 28;16(10):324. doi: 10.3390/membranes16100324 (PMC13706952; doi:10.3390/membranes16100324)
Supplement: Supplementary file 1 [file membranes-16-00324-s001.zip › membranes-4555857-supplementary.pdf]

Supplementary Materials

S1 Reanalysis methods and summary statistics

The data in Table 5 were used without editing, excluding, or supplementing measured responses. Because the responses are tabulated to two decimal places, the calculations reproduce the reported precision rather than unrounded instrument data. The model structure was fixed as [1, A, B, C, BC, A^2, B^2, C^2], with $A = (U - 1.4)/0.2$, $B = pH - 4$, and $C = (t - 400)/100$. No new term-selection procedure was performed. Ordinary least squares used $\beta = (X^TX)^{-1}X^Ty$ with $n = 20$, $p = 8$, and residual degrees of freedom $\nu = 12$.

Let $e = y - X\beta$, $H = X(X^TX)^{-1}X^T$, $h_i = H_{ii}$, and $s^2 = \Sigma e_i^2/12$. $PRESS = \Sigma[e_i/(1 - h_i)]^2$, and predicted $R^2 = 1 - PRESS/\Sigma(y_i - \bar{y})^2$. Internally studentized residuals are $e_i/[s\sqrt{(1 - h_i)}]$. Cook's distance is $e_i^2h_i/[p\ s^2(1 - h_i)^2]$. LOOCV refits coefficients with the same prespecified terms and therefore does not account for original model-selection uncertainty. The coefficient of variation is the residual standard deviation divided by the mean of the 20 observed responses. Adequate precision is the range of the fitted values at the 20 design points divided by $\sqrt{(p\ s^2/n)}$, the average standard error of prediction.

Table S1. Reconstructed model statistics.

Metric	EF	SC
R^2	0.9473	0.9577
Adjusted R^2	0.9166	0.9330
Residual SD	0.0889	0.1734
Fit RMSE using n = 20	0.0688	0.1343
Residual sum of squares	0.0948	0.3607
PRESS	0.3992	1.5333
LOOCV RMSE	0.1413	0.2769
Predicted R^2	0.7781	0.8202
Lack-of-fit F	3.755	3.874
Lack-of-fit p	0.0822	0.0776
Coefficient of variation (%)	3.97	7.37
Adequate precision	22.4	22.2

The six center runs estimate five pure-error degrees of freedom. EF and SC pure-error sums of squares are 0.01515 and 0.05615. Lack-of-fit has seven degrees of freedom. Non-significant lack of fit is not evidence that there is no model misspecification or Faradaic process. Center-point replication does not establish repeatability across fresh component sets.

Table S2. Coefficients and conditional 95% confidence intervals.

Term	EF estimate	EF 95% CI	SC estimate	SC 95% CI
Intercept	2.41555	2.33657 to 2.49453	2.87168	2.71762 to 3.02575
A	0.12548	0.07308 to 0.17788	-0.05542	-0.15763 to 0.04680
B	0.03125	-0.02115 to 0.08365	0.19174	0.08953 to 0.29396
C	0.02113	-0.03127 to 0.07353	0.23002	0.12780 to 0.33224
BC	0.06125	-0.00721 to 0.12971	0.40125	0.26769 to 0.53481
A^2	-0.22809	-0.27910 to -0.17708	-0.36540	-0.46490 to -0.26589
B^2	-0.16269	-0.21370 to -0.11168	-0.50329	-0.60280 to -0.40378
C^2	0.13076	0.07975 to 0.18177	0.10836	0.00885 to 0.20787

S2 Predictions at the selected operating condition

At U = 1.43 V, pH = 4.48 and t = 500 s, the coded point is (0.15, 0.48, 1). For its model vector x_0, the mean-response interval is $\hat{y}_0 \pm t_{0.975,12}\, s\sqrt{[x_0^T(X^TX)^{-1}x_0]}$. The new-run prediction interval uses $\sqrt{[1 + x_0^T(X^TX)^{-1}x_0]}$ instead. These are pointwise intervals under the fixed model and independent, homoscedastic normal-error assumptions, not simultaneous confidence regions for optimal factor settings.

Table S3. Conditional predictions and comparison with the reported initial responses.

Quantity	EF	SC
Predicted response	2.5881	3.3622
95% mean-response CI	2.4970–2.6791	3.1845–3.5399
95% new-run prediction interval	2.3741–2.8021	2.9448–3.7797
Reported initial response	2.54	4.71
Relative difference 100(observed − predicted)/predicted	−1.86%	+40.09%

SC = 4.71 is outside the new-run interval, so it cannot be presented as successful model validation. The intervals apply to a response defined on the same basis as one CCD run. They cannot be narrowed using n = 10 without the actual individual validation values, their aggregation basis, and evidence of independence. Between-component changes and systematic sampling differences are not represented by these intervals.

S3 Residual and influence diagnostics

The maximum absolute internally studentized residual was 2.49 for EF and 2.43 for SC. The maximum leverage was 0.6073. Cook's distance exceeded one for EF run 17 (1.197) and SC run 18 (1.144). Other influential observations include EF run 2 (0.888) and SC run 11 (0.933). These values indicate sensitivity, not grounds to delete valid observations. Run labels are those in Table 5; they are not verified chronological order. Consequently, the run-label panels cannot establish temporal independence or absence of drift.

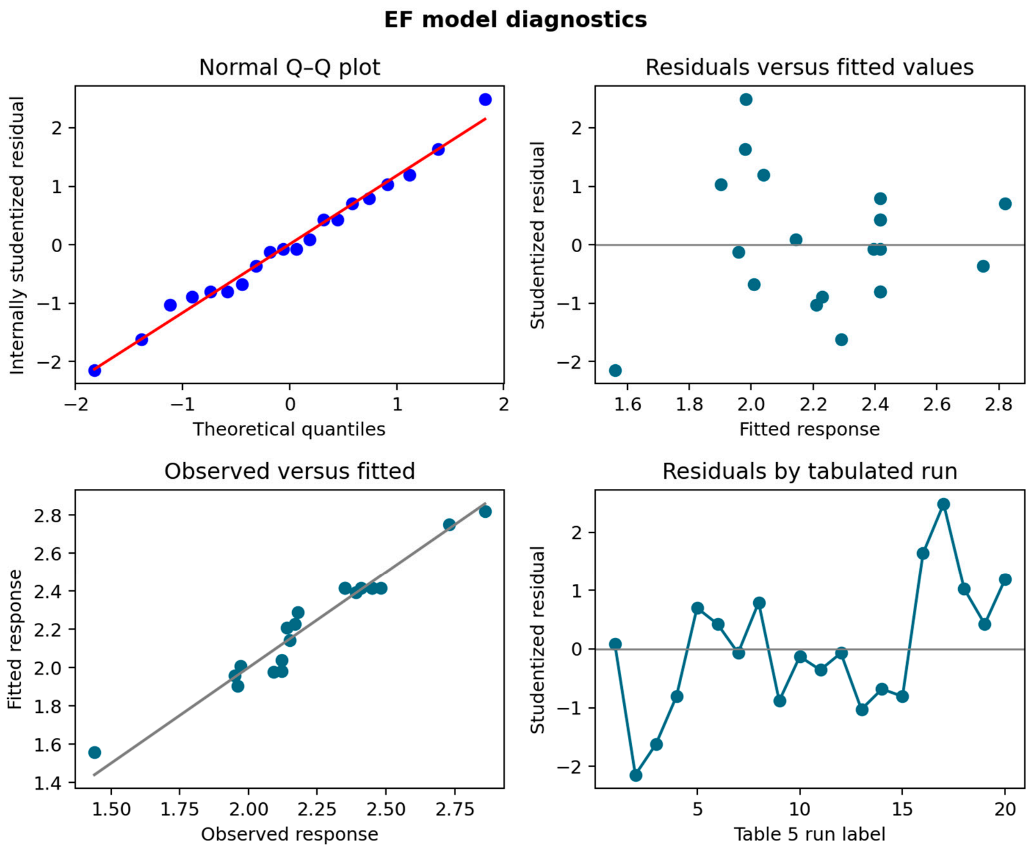

Figure S1. EF diagnostics from the existing CCD data. Q–Q and residual plots describe model residuals; they do not establish external predictive validity or rule out unmeasured mechanisms.

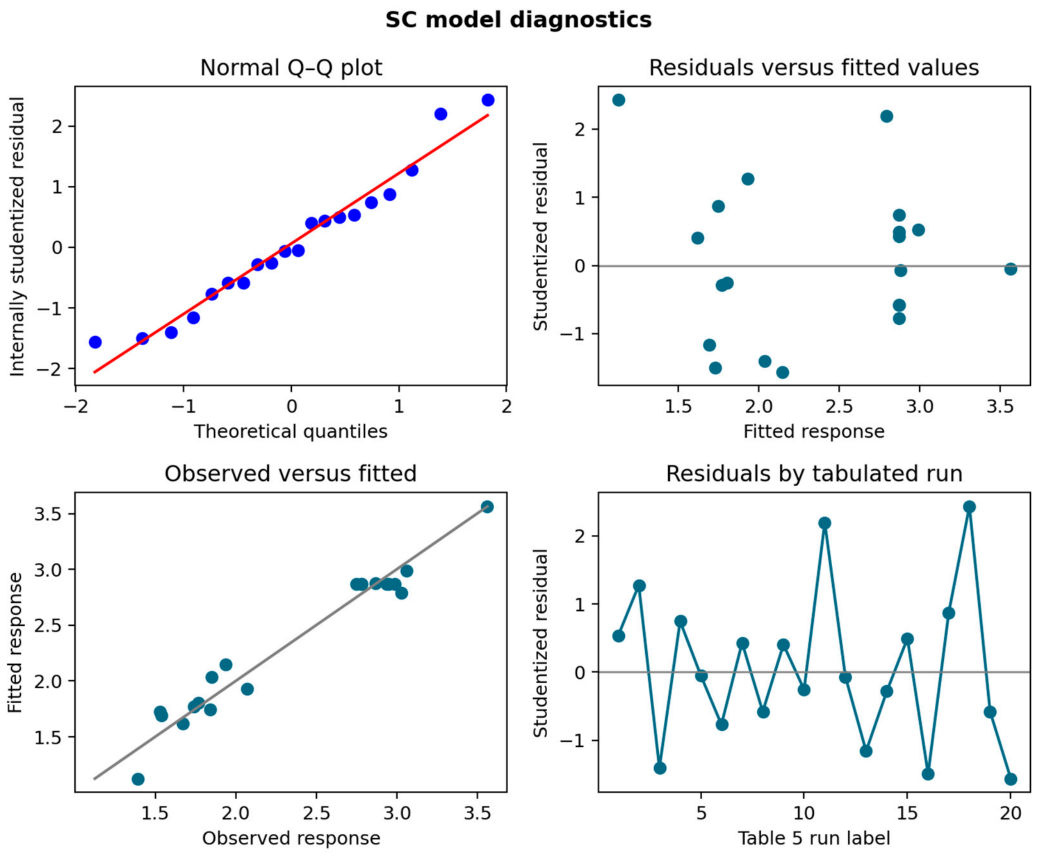

Figure S2. SC diagnostics from the existing CCD data. Q–Q and residual plots describe model residuals; they do not establish external predictive validity or rule out unmeasured mechanisms.

Table S4. Fitted responses and influence measures by Table 5 run label.

Run	EF fit	EF e	EF Cook	SC fit	SC e	SC Cook	h
1	2.144	+0.006	0.001	2.990	+0.070	0.026	0.420
2	1.559	-0.119	0.888	1.931	+0.139	0.315	0.607
3	2.290	-0.110	0.238	2.035	-0.185	0.178	0.420
4	2.416	-0.066	0.016	2.872	+0.118	0.014	0.166
5	2.821	+0.039	0.095	3.565	-0.005	0.000	0.607
6	2.416	+0.034	0.004	2.872	-0.122	0.015	0.166
7	2.416	-0.006	0.000	2.872	+0.068	0.005	0.166
8	2.416	+0.064	0.016	2.872	-0.092	0.008	0.166
9	2.230	-0.060	0.071	1.616	+0.054	0.015	0.420
10	1.959	-0.009	0.001	1.804	-0.034	0.006	0.420
11	2.750	-0.020	0.025	2.791	+0.239	0.933	0.607
12	2.395	-0.005	0.000	2.879	-0.009	0.000	0.420
13	2.210	-0.070	0.096	1.693	-0.153	0.121	0.420
14	2.008	-0.038	0.090	1.771	-0.031	0.015	0.607

Run	EF fit	EF e	EF Cook	SC fit	SC e	SC Cook	h
15	2.416	-0.066	0.016	2.872	+0.078	0.006	0.166
16	1.979	+0.111	0.243	1.727	-0.197	0.202	0.420
17	1.981	+0.139	1.197	1.745	+0.095	0.148	0.607
18	1.903	+0.057	0.204	1.126	+0.264	1.144	0.607
19	2.416	+0.034	0.004	2.872	-0.092	0.008	0.166
20	2.039	+0.081	0.130	2.146	-0.206	0.221	0.420

S4 Desirability calculation and OFAT comparison

The new retrospective calculation used the full-precision reconstructed coefficients. For response k, $d_k = 0$ for $\hat{y}_k \le L_k$, $d_k = (\hat{y}_k - L_k)/(U_k - L_k)$ for $L_k < \hat{y}_k < U_k$, and $d_k = 1$ for $\hat{y}_k \ge U_k$. Limits were (LEF, UEF) = (1.44, 2.86) and (LSC, USC) = (1.39, 3.56). The objective was $D = dEF^{w}\ dSC^{1-w}$; w = 0.5 gives equal importance and unit linear desirability exponents. Bounds were $-1 \le A, B, C \le 1$, with flow 10 mL/min and time ratio 1:1. The factorial search region was narrower than the axial CCD range.

The bounded objective was solved using scipy.optimize.differential_evolution (seed 11, tolerance 10^{-12}, other settings at library defaults). The implementation maximized desirability by minimizing −D. The algorithm and settings describe this revision analysis only; they are not claimed to be those used in the original Design-Expert optimization. No future response at the computed point was experimentally generated.

Table S5. Sensitivity of the model compromise to response importance.

EF weight	U V	pH	t s	EF fit	SC fit	D
0.25	1.4036	4.5405	500	2.5721	3.3824	0.8863
0.50	1.4213	4.4799	500	2.5851	3.3687	0.8575
0.75	1.4382	4.3997	500	2.5941	3.3428	0.8337

The equal-weight solution is near the reported tested condition, whose desirability is 0.8572. The 500 s solution is a boundary result within the chosen search box, not an interior time optimum over the full CCD range. Weighting sensitivity is not a statistical confidence interval for the optimal settings. This calculation optimizes only concentration responses, with no penalty for energy, aging, cleaning or lithium loss.

A residual bootstrap was used to approximate the uncertainty in the location of the equal-weight optimum. For each of 2000 resamples, the residuals of both reduced models were resampled with replacement, inflated by $\sqrt{(n/\nu)} = \sqrt{(20/12)}$, and added to the fitted values; both models were refitted with the same terms, and D was maximized on a grid (step 0.05 in coded units) within $-1 \le A, B, C \le 1$ using the limits above. The 2.5th–97.5th percentiles of the resulting optima were 1.41–1.44 V and pH 4.35–4.65, and the sorption time was at the 500 s bound in all resamples. These intervals are conditional on the model structure and on the CCD component set and do not include between-component variation.

Table S6. EF sorption-time comparison at the common reference voltage and pH.

Time s	OFAT reported EF	CCD model EF at A = B = 0
300	1.43	2.5252
400	1.65	2.4155
500	1.76	2.5674

The positive net model change from 300 to 500 s does not remove the modeled minimum. The short and long axial observations are outside the OFAT range. A concentration-volume explanation remains conditional because adsorption and desorption durations both varied. Component history was not isolated in a matched experiment.

S5 Sulfate accounting

The weighed sulfate additions were 426.9 mg/L $NiSO_4{\cdot}6H_2O$, 152.2 mg/L $CoSO_4{\cdot}7H_2O$, and 91.5 mg/L $MnSO_4{\cdot}H_2O$. Using approximate molar masses of 262.85, 281.10 and 169.02 g/mol, their contributions are 1.624, 0.541 and 0.541 mmol/L. The sum is approximately 2.707 mmol/L or 260 mg/L as SO_4 before acid addition. These are analytical totals, not free SO_4^{2-} activities.

At every pH condition, $S_t = (nNiSO_4 + nCoSO_4 + nMnSO_4 + C_aV_a)/V_{final}$.

The acid volumes added at each condition were not recorded, so St cannot be determined exactly. Because buffering by metal hydrolysis species and HSO_4^- formation can only increase the acid demand, the free H^+ concentration at the target pH gives a lower bound on the added H_2SO_4 of ½·10-pH mol/L, neglecting the H^+ initially present. The corresponding lower bounds on acid-derived sulfate are 0.5, 1.6, 4.8, 48 and 230 mg/L at pH 5, 4.48, 4, 3 and 2.32, i.e., at least 0.2%, 0.6%, 1.8%, 18% and 88% of the salt-derived sulfate; for pH 5.68 the bound depends on the unadjusted feed pH. These bounds are not a substitute for an acid-dose record, and dilution by the added acid was neglected. The 900 mg/L value is the hydrated-reagent sum, not final total dissolved solids.

Metal–sulfate association and sulfate protonation can affect free-ion activities and transport. The cited primary studies [S1, S2] establish association in relevant metal sulfate systems, but their results do not supply a validated speciation calculation for this mixed feed. No association percentage or HCl comparison is claimed.

References

1. S1. Chen, T.; Hefter, G.; Buchner, R. Ion Association and Hydration in Aqueous Solutions of Nickel(II) and Cobalt(II) Sulfate. Journal of Solution Chemistry 2005, 34, 1045–1066. https://doi.org/10.1007/s10953-005-6993-5.
2. S2. Bešter-Rogač, M. Determination of the Limiting Conductances and the Ion-Association Constants of Calcium and Manganese Sulfates in Water from Electrical Conductivity Measurements. Acta Chimica Slovenica 2008, 55, 201–208. https://acta-arhiv.chem-soc.si/55/55-1-201.htm.
3. S3. Hawks, S.A.; Ramachandran, A.; Porada, S.; Campbell, P.G.; Suss, M.E.; Biesheuvel, P.M.; Santiago, J.G.; Stadermann, M. Performance metrics for the objective assessment of capacitive deionization systems. Water Research 2019, 152, 126–137. https://doi.org/10.1016/j.watres.2018.10.074.
